# Complete Protocol and Guidelines for the Implementation and Manufacturing of the Tübingen Palatal Plate—An Interdisciplinary Technical Note on the Tübingen Approach for Infants with Robin Sequence

**DOI:** 10.3390/bioengineering12101063

**Published:** 2025-09-30

**Authors:** Maite Aretxabaleta, Marit Bockstedte, Kathrin Heise, Lisa Theis, Christoph Raible, Katharina Peters, Cornelia Wiechers, Bernd Koos, Christian F. Poets, Christina Weismann

**Affiliations:** 1 Department of Orthodontics, Tübingen University Hospital, Osianderstr. 2-8, 72076 Tübingen, Germany; 2 Centre for Cleft Lip, Palate and Craniofacial Malformations, Tübingen University Hospital, Osianderstr. 2-8, 72076 Tübingen, Germany; 3 Department of Neonatology, Tübingen University Hospital, Calwerstr. 7, 72076 Tübingen, Germany

**Keywords:** pre-epiglottic baton plate (PEBP), intraoral scanning (IOS), personalized medical device, CAD/CAM, upper airway obstruction (UAO), mandibular retrognathia, functional orthodontic appliance, interdisciplinary non-surgical treatment, orthodontic airway plate (OAP), mandibular distraction osteogenesis (MDO) alternative

## Abstract

Robin sequence (RS) is a rare congenital anomaly characterized by micrognathia, glossoptosis, and upper airway obstruction (UAO), often accompanied by a cleft palate. The Tübingen Palatal Plate (TPP), also referred to as the pre-epiglottic baton plate (PEBP), offers a non-surgical, functional orthodontic solution that improves airway patency and feeding by advancing the tongue base. This paper outlines the semi-digital clinical and technical workflow used for TPP treatment at Tübingen University Hospital. The protocol combines intraoral scanning (IOS), computer-aided design and manufacturing (CAD/CAM), and manual refinement for patient-specific appliance production. Practical steps, modifications for special cases and follow-up procedures are detailed, aiming to support clinical implementation at other centres. Based on the published literature and over three decades of experience, the protocol emphasizes safety, quality control, and interdisciplinary collaboration, with practical guidance provided to support implementation in other centres. The potential of digital workflows for data sharing, training, and multicenter collaboration is highlighted, while challenges such as the need for specialized expertise and technical resources are acknowledged. This guideline provides the first comprehensive and reproducible description of the Tübingen approach and aims to facilitate wider adoption of TPP therapy for infants with RS.

## 1. Introduction

**Figure 1:**
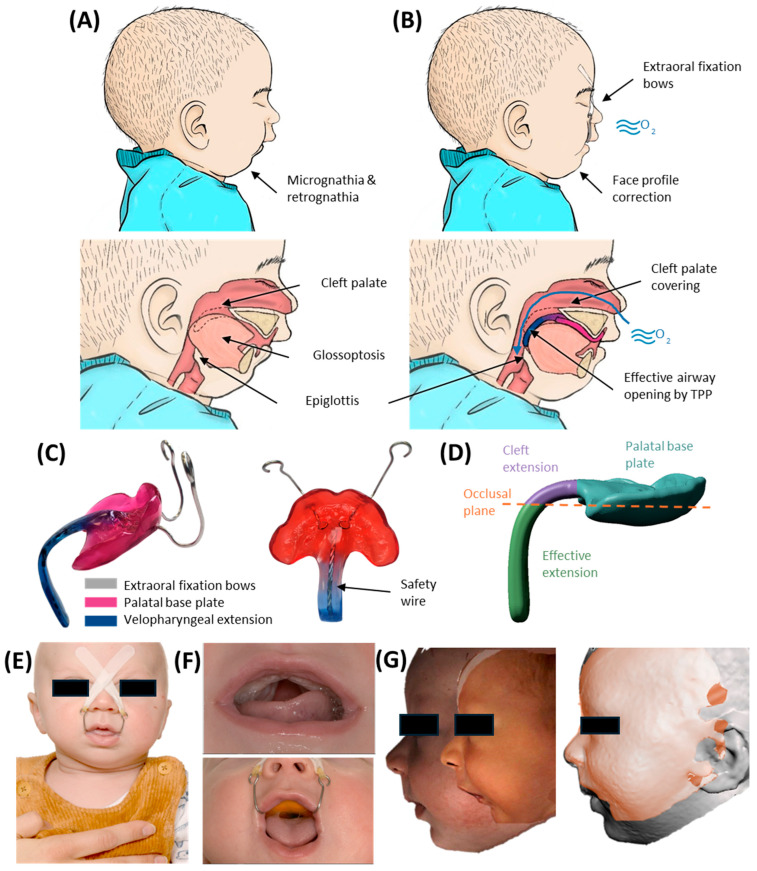
RS and its treatment by the TPP. (**A**) RS characteristics result in UAO. (**B**) Effective TPP therapy leads to airway opening and correction of mandible position. (**C**) Example of an individualized TPP and its parts (Reuse of artwork approved by copyright-holding author (M.A.) [8]). (**D**) Side view of the TPP showing the two parts of the velopharyngeal extension, divided by the occlusal plane into cleft and effective extensions. (**E**) Extraoral frontal image of a patient wearing a TPP, with fixation bows secured using adhesive tapes. (**F**) Intraoral images of a patient with a soft palate cleft without (**top**) and with (**bottom**) the TPP in place [8]. (**G**) Facial profile development of a patient during TPP therapy: shortly after birth and after three months of TPP use.

Robin sequence (RS) is a rare congenital disorder with an incidence of 1/8000 births [1], characterized by mandibular retrognathia and glossoptosis, both leading to upper airway obstruction (UAO) and failure to thrive (Figure 1A). Early treatment is mandatory to prevent long-term sequelae. In 80–90%, RS is associated with a cleft palate (Figure 1F), involving either the soft palate alone or extending to varying degrees into the hard palate [2]. In about half the cases, RS is associated with a syndrome, e.g., Stickler or Treacher-Collins syndrome [1,3].

Among current treatment options, the Tübingen Palatal Plate (TPP)—previously known as the “pre-epiglottic baton plate” (PEBP) [4,5,6,7]—stands out for its efficacy, low invasiveness and ability to induce mandibular catch-up growth [5,8,9,10,11], while directly addressing the root cause of these infants’ symptoms. It consists of a palatal plate with extraoral fixation bows and a velopharyngeal extension reinforced by a safety wire (Figure 1C,E,F) and is designed to widen the pharynx and improve UAO (Figure 1B) [5,12]. As already described in [8], the velopharyngeal extension can be divided into two components (Figure 1D): the “cleft” section and the “effective” section. The “cleft” part depends on the presence and shape of the cleft, as well as the dental technique used to block or spare the cleft area [8]. In contrast, the “effective” extension refers to the portion of the extension located below the occlusal plane, which is the section primarily contributing to opening the airway, as it shifts the tongue base forward, which is typically the main cause of UAO in RS [8].

The velopharyngeal extension extends to just above the epiglottis and rests against the tongue. The pressure from the extension must be sufficient to open the airways while not being excessive, to prevent pressure ulcers and allow functional movements. The appliance also promotes anterior mandibular growth through functional orthodontics, harnessing the body’s muscle activity and natural movements to correct retrognathia and expand the airway —illustrating Roux’s principle that “form follows function” [13]. Two extraoral fixation bows are secured to the patient’s forehead, serving as abutments to absorb the force exerted by the tongue on the extension [14].

The TPP not only alleviates UAO but also enhances the feeding process by ensuring that the tongue assumes a physiological (ventral or anterior) position. Furthermore, the TPP has demonstrated effectiveness in normalizing facial profiles of infants (Figure 1G) [10,11] and improving functional parameters as patients grow [9,15]. This therapy has been successfully administered at the University Hospital Tübingen since 1995 through a collaborative interdisciplinary approach [12,16,17]. However, the complete design and manufacturing process, along with its integration into the multidisciplinary treatment approach, have not yet been comprehensively reported.

This study presents the methodology for the manufacturing of the TPP that has been employed since 2019, when our interdisciplinary team began integrating digital technologies into the routine clinical treatment of RS patients. As interest grows in this minimally invasive approach, we aim to summarize the current knowledge on the manufacturing process to support safe and effective clinical implementation. This document provides a comprehensive, step-by-step protocol covering all stages—from intraoral scanning (IOS) and digital modelling to prototype testing, final appliance fabrication, fitting, and follow-up care. Practical guidance is offered for integrating the TPP approach into centres interested in adopting it, including tips for caregiver instructions, quality and safety checks, and adaptations for non-standard anatomical situations. The study also outlines common complications and their management, highlights the importance of interdisciplinary collaboration, and presents supporting research data. Overall, it serves as both a technical reference and an implementation guide for centres aiming to adopt the TPP protocol.

## 2. Resume of TPP Treatment Workflow

**Figure 2:**
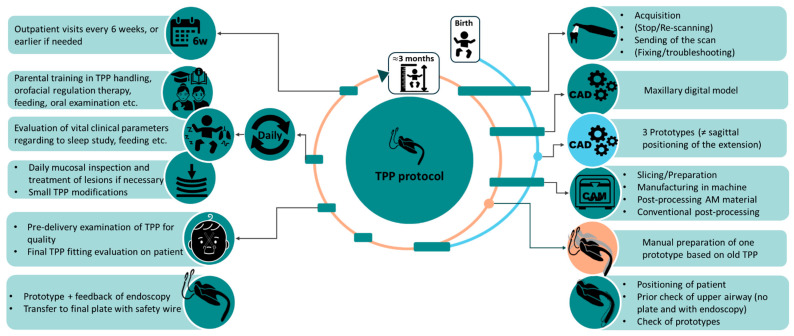
Summary of the TPP workflow detailing steps for initial and follow-up fittings.

Appliance fabrication begins with capturing maxillary dimensions, which was historically performed using alginate impressions. However, since 2019, our group has completely transitioned to IOS of newborns with craniofacial anomalies [18], thereby eliminating the risks associated with alginate impressions [19,20]. A maxillary model is subsequently designed based on the intraoral scan and fabricated using additive manufacturing (AM) techniques [21]. The TPP is initially designed and manufactured using CAD/CAM technologies with a standard velopharyngeal extension, and, based on this digital prototype, subsequently fabricated using conventional methods (Figure 2) [22,23,24,25]. Unfortunately, the intraoral scan only provides maxillary information, leaving the velopharyngeal extension design to the experience of dental technicians and clinicians [8].

The extension’s position is checked via awake fibreoptic laryngoscopy to adjust its length and configuration [8]. Specialized neonatologists and orthodontists assess the positioning as well as the thickness, length and width of the TPP’s velopharyngeal extension, deciding on necessary changes which are then implemented by the dental technician. The device’s effectiveness is confirmed by an overnight study (cardiorespiratory polygraphy), aiming at a mixed-obstructive apnoea index (OAI) < 3/h [7,12]. While endoscopy and sleep studies are essential, invasive imaging techniques are redundant [8,26].

Minor smoothing and corrections of the palatal plate area, such as grinding down some regions to eliminate potential oral mucosal lesions (OMLs), are carried out at follow-up orthodontic visits. Additionally, intensive feeding training and speech therapy based on the Castillo Morales concept are implemented alongside TPP placement [27,28,29,30,31]. Upon successful configuration, feeding training and instructing caregivers in the competent handling of the TPP are the next steps [14], so that patients can ultimately be discharged home. A second TPP may be considered after 2–3 months, contingent on a sleep study still indicating an OAI > 3/h (following 3 days without a plate to rule out carry-over effects) [7].

Treatment typically continues until 6–7 months of age, during which most patients require two, occasionally three, TPPs to accommodate head growth [7]. Initial plate fitting is more time-consuming and demanding for both patients and parents due to the longer configuration process. With the second plate, families are usually familiar with the therapy. A new intraoral scan and updated maxillary model—produced via AM—are obtained, but no new digital prototype is needed, as the previous TPP serves as a template for conventional fabrication. This streamlines the process and shortens the time from scanning to appliance delivery compared to the initial fitting.

**Figure 3:**
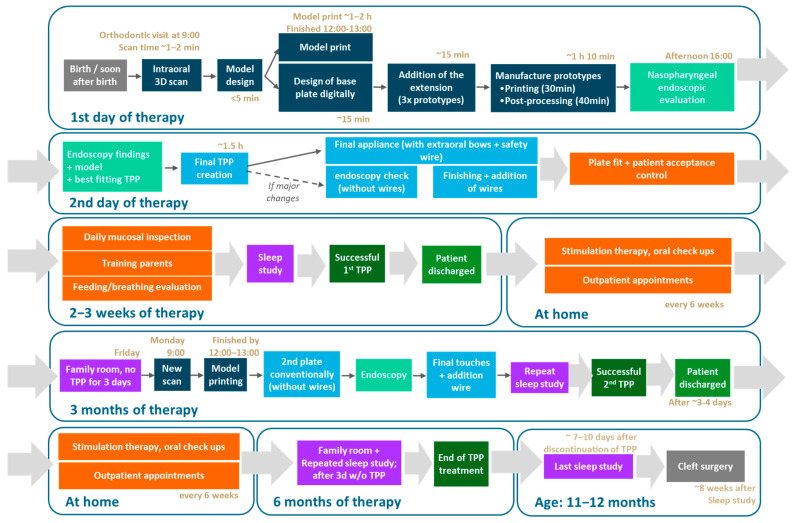
Exemplary interdisciplinary workflow implemented in our centre with the most common time approximations for each step. The workflow is shown for the most common scenario of a patient requiring two TPP.

Figure 3 presents an example of the complete protocol implemented in our facility, focusing on the successful in-house design and fabrication of the personalized appliance, along with the typical timeline for each step of the implementation workflow.

## 3. Prototyping Stage for the First Patient-Specific TPP

### 3.1. Digital Manufacturing Through CAD/CAM

**Figure 4:**
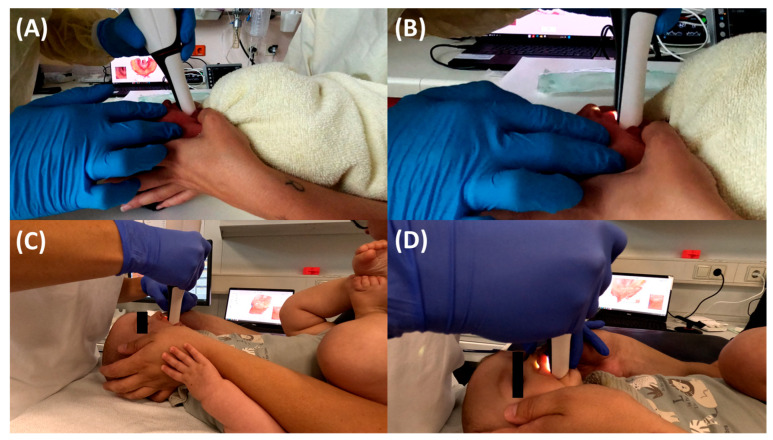
Scanning procedure in patients with RS, showing patient and laptop positioning. (**A**,**B**) A newborn swaddled in a blanket and held by the caregiver. (**C**,**D**) Scanning of a 5-month-old patient while being held by the caregiver.

TPP treatment begins with IOS, ideally directly after birth. In infants with lower weight, the procedure may need to be delayed due to insufficient oral aperture [18]. Infants with severe retrognathia may benefit from early orofacial regulation therapy to improve mouth opening. Unlike traditional impressions, IOS eliminates the need for fasting or sedation; however, it is recommended to wait at least one hour after feeding before performing the procedure [18]. Infants are scanned in the supine position (Figure 4), with the head oriented towards the clinician to facilitate oral access, while the clinician positions the laptop in front for better control [18]. Immobilization of the patient, if necessary, is achieved through swaddling in a blanket and with the assistance of a parent, effectively preventing movement or interference by the patient.

**Figure 5:**
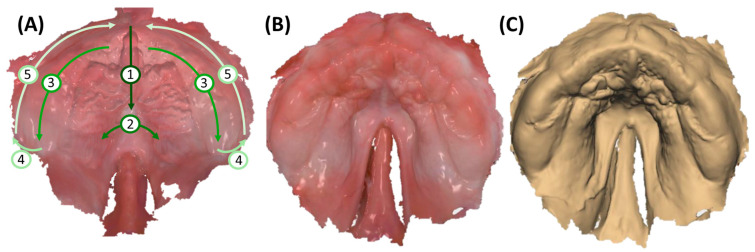
IOS of maxillae for infants with RS. (**A**) Example of a maxillary soft palate cleft and the intraoral scan protocol based on [18], showing sequential scanning steps (Steps 1–5). (**B**) Example of another maxillary scan for an RS patient with a U-shaped cleft (scan time in this case was 1 min 48 s). (**C**) Same image without colour and texture information to allow for proper structure identification.

Initially, towards the end of 2018, we used the Trios 3 scanner, which was updated to the Trios 4 in early 2022 (3Shape A/S, Copenhagen, Denmark). These scanners, employing confocal scanning technology, feature a larger built-in tip and have proven effective for routine clinical use. Over the past more than six years, successful scans have been performed in a large number of patients with various craniofacial disorders [18]. A consistent scanning protocol is applied [18] (Figure 5). Challenges during scanning include a restricted mouth opening in some RS patients due to unique anatomical conditions and recording of the posterior tuber region. This can be addressed by tapering the scanner head to capture the most posterior region.

Infants’ anatomy and compliance influence the ability to obtain an appropriate scan. Scanner operator training and software/computer issues, such as slow-running systems or updates, may also affect scanning. Despite automatic artefact removal by the scanner software, artefacts are sometimes unavoidable and may require manual correction [32]. This is typically addressed using the “cut” function within the software to manually remove unwanted scan data. In challenging cases, mesh editing freeware like Meshmixer (Autodesk Inc., San Rafael, CA, USA) can be used [32]. Post-processing or rescanning may not always yield optimal results. Posterior tuber region images are challenging but not vital for TPP design. Scans without this region are sufficient, but about two-thirds of the alveolar arch should be visible, so that an appropriate palatal base plate that avoids rotation of the appliance can be found.

**Figure 6:**
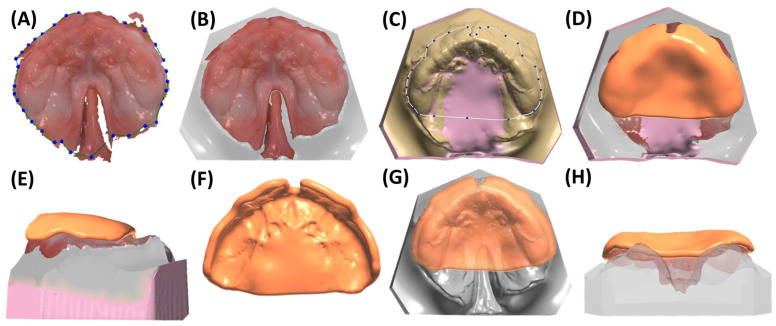
Intraoral scan-based creation of the maxillary model and TPP base palatal plate: (**A**) Scan region delineation. (**B**) Digital model creation from the intraoral scan. (**C**) Digital wax addition and spline definition for the palatal base plate contour. (**D**) Base palatal plate creation on the digital model, based on the defined spline (**C**) after smoothing. (**E**) Side view of the flat occlusal plane on the base palatal plate. (**F**) Detailed negative image of the palatal side. (**G**) Top view of the final appliance within the patient’s maxilla. (**H**) Side view of the final appliance positioned on the maxilla, leaving the cleft area uncovered.

Scan data are saved as STL (standard tessellation language) for shape or PLY (polygon file format) and .dcm (digital imaging and communications in medicine) for shape and colour, automatically sent to Ortho System (3Shape A/S, v.1.11.2.0). Dental model creation involves defining occlusal and sagittal planes (selecting regions corresponding to tooth regions of 16, 11–21, and 26), adding a spline to delimit the region of interest (discarding artefact regions) (Figure 6A), and optimizing socket dimensions based on the obtained scan (Figure 6B). Minimizing dimensions is crucial to reduce time and material costs for posterior model printing. Approximately 5 mm of additional model area is added for posterior side, behind the tuber region, for later support in conventional manufacturing; for incomplete tuber region recordings, extra millimetres are added. The model is edited with a smoothing tool to remove artefacts. Subsequently, it is additively manufactured using stereolithography (SLA) with Preform V.2.0. software and a Form3B SLA machine (Formlabs, Sommerville, MA, USA). Model V3 material (Formlabs) is employed, with a layer height of 100 µm for optimal accuracy and efficiency [25]. Post-processing includes a 20 min 99% IPA (isopropyl alcohol) wash, air drying, and a 30 min post-curing at 70 °C in Formcure (λ = 405 nm, Formlabs), with support structures removed after complete polymerization.

Simultaneously, the patient-specific TPP is created using Dental Manager software (3Shape A/S, v.2.103.1.2). The individual impression tray module is employed for the maxilla, where the previously designed model is imported in .dcm format. Digital wax is used to block specific regions (Figure 6C), creating space for the extension and accommodating cleft width reductions. When adding wax, a thickness of approximately 2–3 mm is recommended, as the TPP base plate should be positioned about 2–3 mm above the bottom of the cleft palate. This spacing ensures adequate tongue room, supporting physiological function and allowing for unrestricted dorsal pharyngeal movements during swallowing. Additionally, a flat, plane-like wax layer should be obtained to ensure that the base plate conforms to the flat shape of the cleft region (Figure 6H). The program allows for a maximum wax layer thickness of 4 mm. For deep palatal clefts, the base model can be modified by adding model material [32]. Strategic wax placement also addresses areas lacking information or showing minor injuries. The software automatically covers undercuts with wax, smoothing sharp edges. A spline delimiting the TPP base shape is set on the wax model, following a linear pattern towards the posterior end of the tuber region (Figure 6C). The software generates a plate of 2 mm thickness, which can be smoothed with software tools for the removal of any artefactual regions from spline-to-plate computation (Figure 6D). Presented model and plate protocols have also been detailed in previous studies [21,22,32].

**Figure 7:**
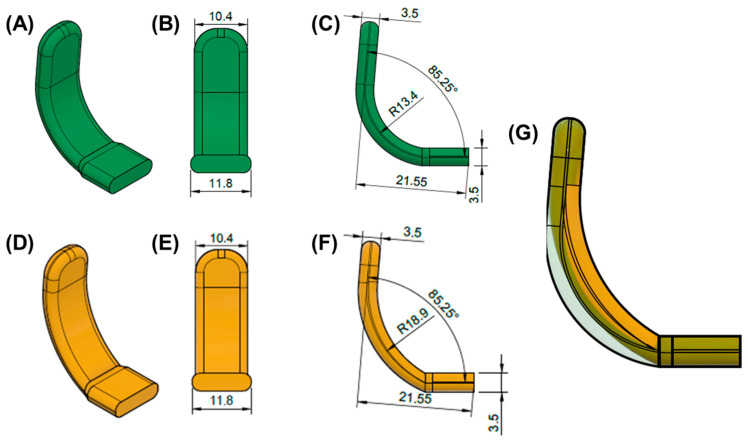
Key measurements (mm) of standard TPP velopharyngeal extensions: (**A**) Isometric view. (**B**) Front view. (**C**) Lateral view of the extension for children with a cleft palate. (**D**) Isometric view. (**E**) Front view. (**F**) Lateral view of the extension for children without a cleft. (**G**) Superimposition of the two standard TPP velopharyngeal extensions.

Once the base plate is established (Figure 6E–H), the velopharyngeal extension is attached. A standardized extension, developed and refined by experienced dental technicians over 30 years, serves as the basis. Originally crafted manually, the shape underwent digitalization and redesign using CAD software (Fusion360, v.2603.1.52, Autodesk Inc.) to create a standardized digital file of the velopharyngeal extension. This common extension maintains consistency across most patients during the prototyping stage. The “cleft extension” portion of the velopharyngeal extension has two variants: one designed for patients with a cleft (Figure 7A–C) and another for patients without a cleft or with a small soft palate or uvular cleft only (Figure 7D–F). The latter version features a greater curvature radius or a flatter appearance in the cleft region (Figure 7G). In these cases, more palatal structures are present, resulting in less space for their movement compared to patients with larger clefts. To allow proper physiological movement of the posterior palate, additional space must be provided by the extension, resulting in a flatter upper region for this patient group. Both versions share a characteristic curved design with rounded edges and a blunt, rounded tip that securely attaches to the palatal plate base. For detailed specifications, refer to the technical drawings provided in the Supplementary Materials, where also .stl files of the respective Tübingen standard extensions can also be found.

**Figure 8:**
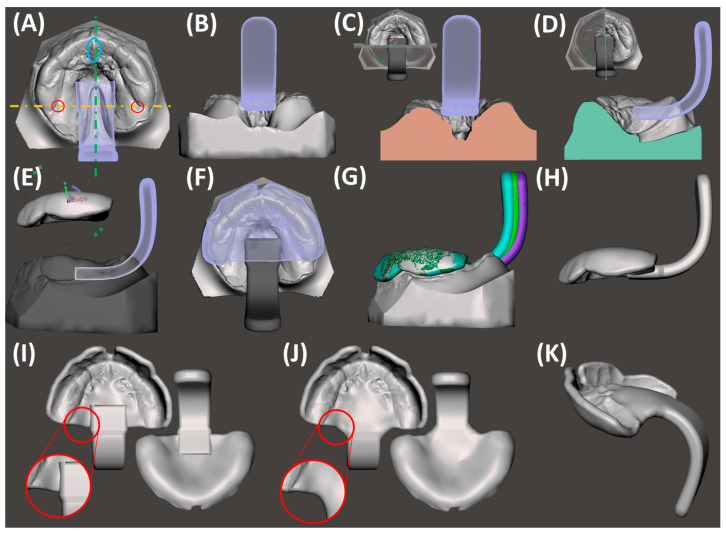
Velopharyngeal extension positioning and attachment for patient-individualized TPP prototypes using Meshmixer. (**A**) Positioning of the extension on the maxillary model, aligning the extension’s design corner with the posterior maxillary gingival landmarks (red) and the middle of the maxilla using the papilla incisive (blue) as a reference, creating a transversal reference by the first (orange) and sagittal by the second (green). (**B**) Horizontal view showing the depth of the extension in the maxillary model. (**C**) Close-up view of the depth and positioning of the extension using a transversal cutting plane through the digital model. (**D**) Close-up view of the depth of the extension position using a sagittal cutting plane through the digital model. (**E**) Addition of the base plate to the digital model and standard extension. (**F**) Alignment of the base plate to the digital model and standard extension setup, ensuring the negative of the palatal base aligns with the model. (**G**) Construction of extensions in 2 mm increments in the sagittal direction: base design (green, no. 2), anterior design (blue, no. 3), and posterior design (purple, no. 1). (**H**) Lateral view of the final base plate and extension setup. (**I**) Top and bottom views of the plate prototype before refining connecting structures. (**J**) Top and bottom view of the plate prototype after refining connecting structures, smoothing, and enlarging areas. (**K**) Finished TPP prototype.

For the extension addition, the model file (without digital wax), the base plate, and the standard extension are imported into Meshmixer software (v.3.5.474). To obtain the correct sagittal positioning of the extension, it is initially centred in the middle of the cleft region, aligning it with the papilla incisive landmark (Figure 8A). For highly asymmetrical maxillae, either because of an abnormally shaped alveolar ridge, papillae, or cleft, the cleft space is used as a reference, where the extension will be positioned in the middle. For transverse positioning, the most posterior points of the gingival grooves on both sides are used as visual reference lines, aligned with the anterior extension guideline (Figure 8A). This becomes challenging if only two-thirds of the tuberosity are scanned or one side is entirely missing. Although two-thirds of the tuberosity are theoretically sufficient to fabricate the TPP and prevent intraoral rotation, it complicates precise extension positioning. In such cases, the posterior gingival reference must be estimated, requiring approximation of its real-life location.

Considering height positioning, alignment with the posterior gingival landmarks is ensured (Figure 8B,C). In the lateral view, the inner surface of the extension should be perpendicular to the occlusal plane (Figure 8D). The plate is positioned on the model, defined as “target”, and matching surfaces on the plate (negative of the model) are selected, limited to those not covered by digital wax. These regions are used for the “Align to Target” function, ensuring an optimal fit (Figure 8E,F). Once positioned, the palatal plate and extension are joined using the “Boolean union” function (Figure 8H). The newly fused part is exported as .stl format. Two additional prototypes are created by translating the extension 2 mm posteriorly and anteriorly in sagittal direction (Figure 8G). The “Boolean union” function is applied again to combine extension and base plate. Combined TPP prototypes (Figure 8H) undergo refinement of connecting structures, smoothing of surfaces, and enlargement of certain areas for structural safety (Figure 8I,J) to achieve the final prototype (Figure 8K), which is then exported as STL. The TPP with the most posterior extension is named prototype 1, whereas the middle and most anterior ones are labelled prototypes 2 and 3, respectively.

The three prototypes are manufactured using a Direct Light Processing (DLP) printer (Solflex 170, W2P Engineering GmbH, Vienna, Austria) with a 100 µm layer height and Medical Device Regulation (MDR) Class I material in blue (Freeprint Tray, DETAX GmbH and Co. KG, Ettlingen, Germany), following recommendations from a previous study [24]. Despite being initially intended for creating 3D-printed and patient-individualized impression trays, the Class I material proved safe for prototype manufacturing and endoscopic control, with its green colouring offering good contrast to the pharyngeal mucosa [23,24]. Additionally, DLP technology provides simultaneous and fast manufacturing of the three TPP files. A 100 µm layer height offers optimal accuracy for the intended application, shortening manufacturing time [25]. The printing job for the DLP machine is prepared using Autodesk Netfabb Premium 2021 (Autodesk Inc., San Rafael, CA, USA).

**Figure 9:**
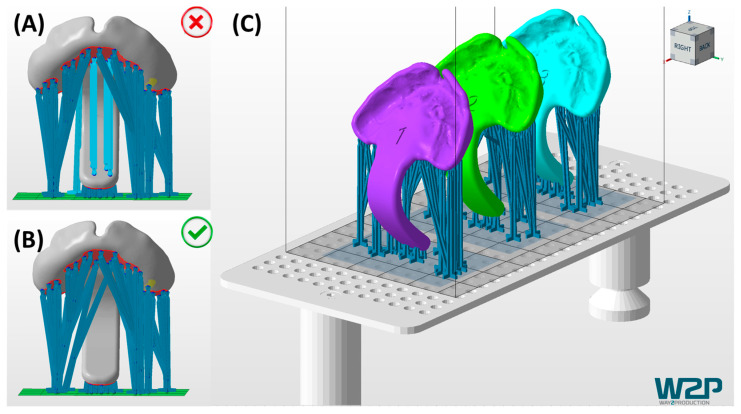
Preparation of the manufacturing job, or the slicing step for DLP manufacturing of multiple prototypes, is carried out in Autodesk Netfabb. Careful placement of supporting structures is crucial to avoid unnecessary contact or placement in the extension region, as illustrated in (**A**) incorrect example, whereas (**B**) demonstrates the correct approach. Each part is labelled with an engraved number (1, 2, 3) and positioned on the building platform, as depicted in (**C**). Manufacturing time for three prototypes in this specific example was 1 h 7 min for 506 layers (layer height of 100 µm).

TPP prototypes are engraved with their respective numbers (1–3) added in the cleft region (Figure 9), enabling prototype distinction. They are inclined with the palatal side facing away from the building platform to prevent surface defects from support structures (Figure 9). Support structures (VOCO GmbH, Cuxhaven, Germany) are employed with special attention to avoiding unnecessary placement in the extension, minimizing defects during removal. Base-supporting structures landing on the extension are shifted to the side to prevent unnecessary support contact (Figure 9A,B). As the extension is aimed to be as intact as possible to ensure mechanical stability, avoidance of cracks or processing errors derived from the removal of supporting structures is indispensable [24].

**Figure 10:**
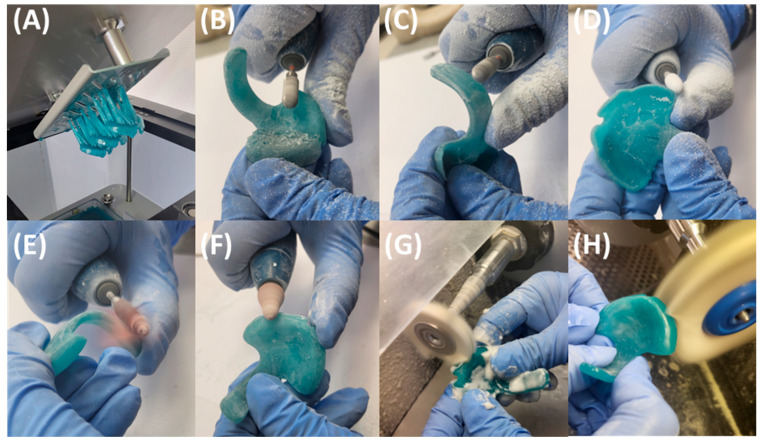
Manual post-processing steps for TPP prototypes after AM: (**A**) Removal of supporting structures. (**B**) Removal of marks from support structures, with the top portion ground down, revealing supporting marks in the unprocessed bottom portion. (**C**) Elimination of layer–staircase-effect–induced roughness throughout the TPP prototype (except for the occlusal side). (**D**) Reduction of the palatal plate rim. (**E**) Initial polishing with sandpaper. (**F**) Subsequent polishing with a fine polisher. (**G**) First mechanical polishing using pumice powder and a buffing wheel. (**H**) Second mechanical polishing with polishing paste and a buffing wheel.

After the manufacturing process, the prototypes undergo post-processing for final polymerization of the resin-based parts (Figure 10A), following the manufacturer’s instructions. This involves a two-step ultrasound bath cleaning procedure in 99% IPA for 3 min each, interrupted by drying with compressed air. Subsequently, support structures are removed, avoiding forced breakage of the non-polymerized appliances. The polymerization process is completed through a two-step post-curing process in Otoflash G171 (λ = 280–700 nm, NK-Optik GmbH, Baierbrunn, Germany), utilizing 2000 flashes in a nitrogen atmosphere. Between curing cycles, samples are rotated and allowed to cool for 2 min with the chamber lid open. The process concludes with manual finishing of the parts (Figure 10). Marks from support structures and roughness from manufacturing layers are ground down throughout, except for the occlusal side of the palatal plate. The vestibular palatal plate rim is reduced to 1.5 mm for a more comfortable fit between the lip and alveolar ridge. Two-fold polishing steps are performed: first using sandpaper of grain size 120, then with a fine polisher (REF 1510/12, KerrHawe SA, Bioggio, Switzerland). Sharp edges are removed by low-grain sandpaper polishing. Surface polishing is necessary to reduce the roughness inherent to vat polymerization CAM technologies like DLP. This is crucial for the velopharyngeal extension, as it ensures a smooth sliding against the pharyngeal mucosa during endoscopic fitting, avoiding mucosal irritation and improving patient comfort. However, the occlusal area of the plate remains unprocessed, aside from minor grinding of potential sharp edges, retaining its textured layering effect and promoting better device adherence to the maxillary mucosa, as sanding down compromises accuracy and may result in a poorly fitting plate [25]. Finally, two-step mechanical polishing (3000 rpm) is executed using powdered pumice, a soft buffing wheel, and polishing paste. No extraoral fixation bows or safety wires are attached to the prototype.

**Figure 11:**
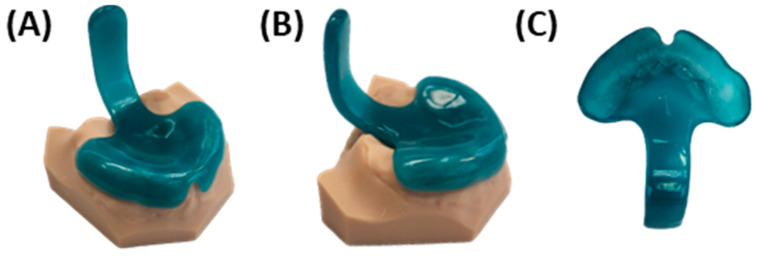
Final TPP prototype in isometric (**A**) and lateral view (**B**) with the corresponding maxillary model, along with the bottom view (**C**) of the occlusal side of the TPP, labelled with engraved number 1.

The finalized TPP prototypes must meet minimum quality standards for their subsequent use in endoscopy (Figure 11). In terms of manufacturing, adherence to MDR guidelines and the manufacturer’s specific material processing instructions is crucial, as previously detailed. This ensures the material’s properties under applied forces remain appropriate [24]. Additionally, the produced prototypes should be free of defects arising from both the manufacturing process (e.g., microcracks, bubbles) and manual post-processing steps.

### 3.2. Endoscopic Exploration of the Prototypes

**Figure 12:**
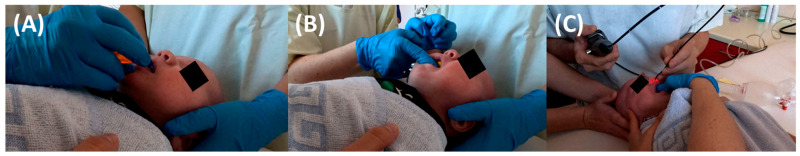
Endoscopic evaluation of the prototype and positioning of the clinicians. (**A**) Placement of the TPP prototype. (**B**) Lightly pressing the base palatal plate against maxillae with a finger to ensure proper attachment and positioning. (**C**) While still lightly holding the TPP in place, evaluation is performed by awake nasopharyngeal endoscopy.

It is advisable that infants are not fed for at least two hours prior to endoscopic evaluation of TPP prototypes to minimize the risk of vomiting and aspiration of milk [7]. Endoscopy is performed using a flexible fibreoptic nasopharyngeal endoscope (C-MAC^®^, Monitor 8403 ZX, Karl Storz, Tuttlingen, Germany). Following the same protocol as used for IOS, the patient is again swaddled in a blanket in supine position (Figure 12). Endoscopy is performed while the patient is awake and without sedation to allow assessment of pharyngeal function under normal muscle tone and to evaluate how the TPP interacts with the anatomical structures during physiological conditions [33,34,35]. Before assessing the fit of the TPP extension, the pharyngeal and posterior oral cavity are examined to exclude other protuberances, mucosal abnormalities, or malformations (e.g., laryngomalacia). This examination may also help assess the severity of UAO, classified according to Sher et al. (Figure 26) [36]. In cases with excessive saliva or mucus that may hinder proper endoscopic evaluation, 40% glucose solution (Glucose 40) can be administered to stimulate swallowing.

**Figure 13:**
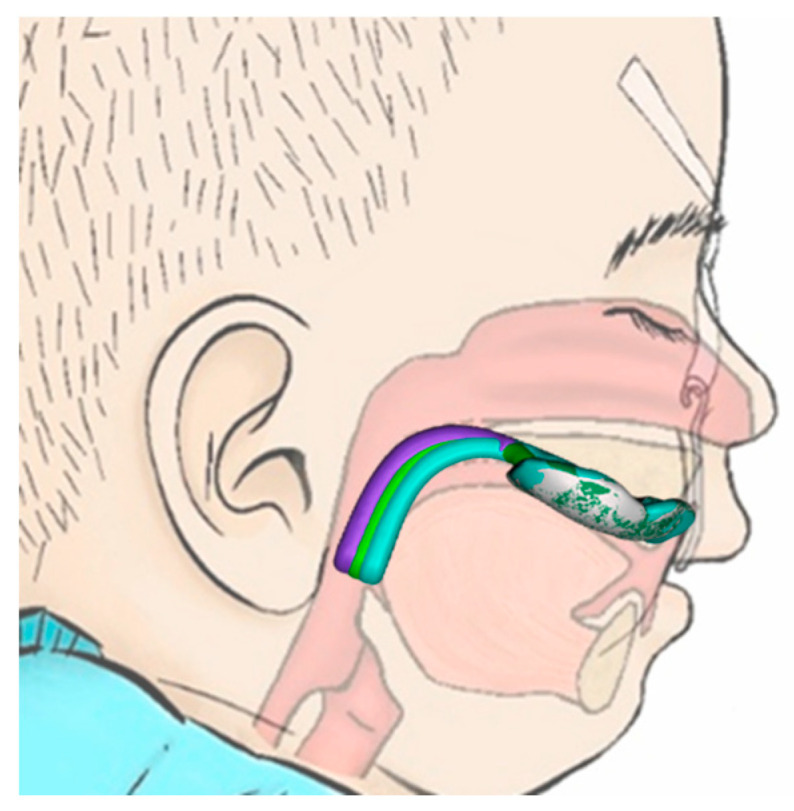
Scheme showing a patient with TPP extension configurations in 2 mm sagittal increments: base (green, no. 2), anterior (blue, no. 3), and posterior (purple, no. 1).

After the initial assessment, the prototype fit is evaluated. The patient lies in a supine position with the head in neutral alignment (the so-called “sniffing position”). The clinician performing the endoscopy positions the patient’s head close to themselves, while the person inserting the TPP stands laterally. The prototypes are then tested sequentially in the order of their engraved numbers (1 to 3) until an optimal fit is achieved (Figure 13). This ensures that the first prototype enters the pharyngeal cavity with minimal resistance and pressure on the tongue, enhancing procedural safety.

The initial prototype is first inserted without the endoscope in place (Figure 12A,B). After positioning the prototype, the tongue must be visible in an anterior position. Patients should not gag or retch upon insertion of the plate, as this indicates that the extension is too long and needs to be removed immediately—without endoscopic evaluation. In this case, extensions of all prototypes are shortened by a few millimetres before trying them again. Fortunately, this happens only rarely when using the standard length. If the response persists after shortening the extension, further reductions are required. This can be performed at the bedside by the orthodontist using a portable manual grinder.

**Figure 14:**
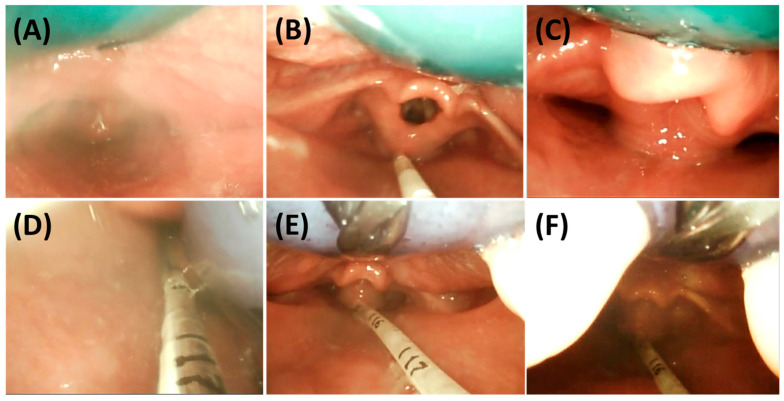
Exemplary endoscopic findings with the TPP prototype (green, (**A**–**C**)) and final TPP (blue, with safety wire, (**D**–**F**)). (**A**) The velopharyngeal extension is too short, allowing the tongue to protrude into the airway. (**B**) The extension length is appropriate, supporting the epiglottis in an upright position and advancing the tongue to open the upper airway; the gastric tube (white) is visible at the bottom of the image. (**C**) The extension is too long, hindering epiglottic movement. In anatomically challenging cases, the definitive TPP may be required for accurate fit assessment. (**D**) The extension is positioned too far posteriorly, pressing against the pharyngeal wall. (**E**) The extension is too short and steep, causing the tongue to protrude alongside the appliance and leading to laryngeal collapse. (**F**) Endoscopic inspection of the palatal cleft shows a narrow cleft with a bifid uvula; thus, the upper part of the extension was reduced in width to allow physiological movement of the soft palate.

Once the first prototype is inserted, the orthodontist ensures the correct positioning of the prototype and stabilizes the palatal base against the maxilla using a finger (Figure 12B,C) to prevent movement and ensure proper positioning during evaluation of the extension. Two main parameters are assessed: first, the extension is evaluated for its ability to visibly open the airway sufficiently without impeding the movement of the epiglottis. In addition, the behaviour of the pharyngeal region and epiglottis is closely monitored during breathing, crying, swallowing, or coughing to assess interaction with the velopharyngeal extension. Second, the distribution of forces exerted by the extension along the tongue is assessed. Unequal, excessive, or insufficient pressure is often apparent during insertion by the clinician and can be confirmed laryngoscopically. If forces are insufficient or the extension is not positioned anteriorly enough, it may fail to fully open the airway or adequately move the tongue base forward (Figure 14D). Conversely, excessive force may make insertion difficult and lead to visible tongue protrusion around the extension (Figure 14E). If the first prototype exerts insufficient pressure, the second prototype is tested, followed by the third if necessary. This approach substantially reduces the risk of a TPP prototype design where the extension is positioned too far anteriorly, which would impede its placement in the pharynx, further reducing the need for redesigning and rescheduling another endoscopy [7]. Excessive force or an overly anterior design is likewise undesirable, as it generates strong counterbalancing forces on the maxilla, leading to adverse effects such as OMLs and deep dents, described in more detail below. Furthermore, this sequential approach (steps 1 to 3) using different configurations enables efficient fitting within a single endoscopy session.

The plate’s sagittal position is first assessed to ensure adequate resistance against the tongue. Its length is then evaluated relative to the tongue base and epiglottis (Figure 14D–F). While overly long extensions can be easily adjusted bedside, shorter extensions are more difficult to correct (Figure 14A–C), requiring laboratory modification and a second endoscopy session. The required modification should be approximated and prepared in the dental laboratory. An optimal length of the velopharyngeal extension ensures that it extends to just above the epiglottis with the head in a neutral position, ensuring full epiglottic mobility. It should also be positioned slightly above the vallecular region to prevent OMLs (Figure 1B and Figure 14B) [7]. Additional minor adjustments, such as curving the distal third of the extension to position it between the tongue root and the epiglottis, may also be determined at this stage. In a controlled setting, thickness may be reduced but should not be <3 mm. In certain cases, it is important to ensure the extension is centrally positioned to prevent asymmetrical tongue bulging, which may result from an improper TPP design or, more commonly, from patient-related asymmetry. The initial reduction in the cleft extension will be approximated bedside and then secured with the safety wire for the final TPP. At no point should stability be compromised by excessive material removal, as this could pose a risk—even in a controlled clinical setting—of breakage and aspiration. However, following the presented protocol, such incidents have never occurred at our centre.

In cases involving a narrow palatal cleft with large uvula cleft segments, a narrower connection between the extension and its palatal base may be required. This adjustment can be easily performed at the bedside during endoscopy to allow physiological soft palate movements during swallowing and improve patient comfort (Figure 14F). However, this should only be performed once the correct prototype has been confirmed. The width of the extension may be reduced, but this reduction should never exceed half the original width to preserve stability. Extension thickness cannot be altered at this stage and may only be adjusted in the dental laboratory once the safety wire is in place.

All modifications requiring laboratory adjustments must be clearly documented to guide the dental technician in customizing the final TPP. In rare cases, such as anatomical asymmetries in the maxilla or pharynx, lateral adjustments of the extension may be necessary. An internal form employed for TPP modifications is provided as a Supplementary File.

## 4. Transfer of Prototype to Final Appliance

**Figure 15:**
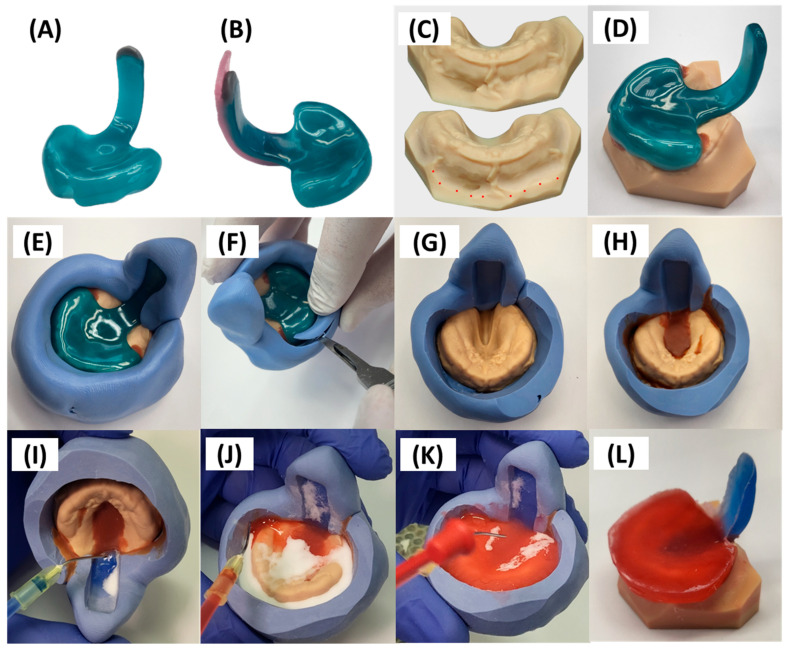
Process for transitioning the CAM-manufactured TPP prototype into the final TPP. (**A**) Lengthening of the prototype’s extension for conventional manufacturing through wax modelling. (**B**) Wax modelling of the prototype for posterior extension movement. (**C**) Folds generated from the scan-to-socket connection before and after their elimination (indicated by red dots). (**D**) Lateral view of TPP placement and securing with wax on the model. (**E**) Application of duplication silicone on the prototype–model setup. (**F**) Removal of edge silicone to allow for the extraction of the prototype. (**G**) Model-silicone assembly. (**H**) Model–silicone assembly with wax blocking on the palatal side and undercuts. (**I**) Polymerization of the extension. (**J**) Polymerization of the base palatal plate. (**K**) Addition of final layer of powder. (**L**) Rough shape of the polymerized TPP on the corresponding model.

First, any changes identified during endoscopy should be transferred to the best-fitting prototype. Feedback for optimizing the TPP extension focuses on potential modifications in length and sagittal positioning. Minor adjustments—such as material removal from the posterior surface or shortening of the extension—can be performed bedside during the endoscopy session by grinding. In contrast, adding material requires modifications performed in the dental laboratory. If elongation of the extension is necessary, sculpturing wax (Thowax grey sculpturing wax, Yeti Dentalprodukte GmbH, Engen, Germany) is placed at the distal tip of the extension (Figure 15A). To move the extension more posteriorly sagittally, material is applied to the posterior surface of the extension using wax plates (Figure 15B; modelling wax, Pluline Rosa, Pluradent GmbH & Co. KG, Offenbach, Germany). In contrast, if the extension needs to be placed more anteriorly than the third prototype, this is achieved by adding a wax plate to the front, followed by selective grinding of the extension. The number of wax plates employed depends on the degree of sagittal movement needed. The same process will be applied for minor adjustments in specific areas, such as advancing the lower third of the extension to create a more pronounced so-called “C-shape”—a common modification to spare the epiglottis and facilitate its smooth movement. These modifications allow precise simulation of the optimal extension shape prior to final fabrication.

After applying these changes to the TPP prototype, it must be combined with the corresponding printed maxillary model. In some instances, the model folds must be ground to ensure a proper fit of the TPP (Figure 15C). Some of these folds represent artefacts rather than true anatomical structures, arising from scan data processing and the design of the base socket. In addition, the relatively large scanner head causes tension in the mucobuccal folds during the scanning procedure, which may lead to their distorted appearance in the scan and result in further inaccuracies in this region of the model. Furthermore, minor shrinkage of the TPP material can further affect the fit, making adjustments necessary. Smoothing these regions also supports the creation of a seamless finish at the TPP margins. Special care is taken during removal to protect structures corresponding to labial and buccal frenula. Once both the prototype and the model are prepared, the prototype is fitted onto the model and securely positioned with 3–4 points of adhesive wax (Supradent-Wachs, Chemisches Dental-Labor Oppermann-Schwedler, Bonn, Germany) (Figure 15D).

Next, the current model-prototype setup is encased in a silicone duplication contour (Figure 15E) with a 1:1 mixing ratio, using approximately two spoons each of catalyst and base (Blue eco lab putty, A-silicone based 86 shore A, ref 02467, DETAX). The configuration is allowed to harden for 4 min following manufacturer’s instructions before proceeding. Sufficient silicone thickness around the TPP extension is essential to ensure stability during subsequent manipulation. Inadequate thickness may compromise the mould’s integrity and result in unintended shifts in the extension’s position, potentially altering its orientation irreversibly. Furthermore, applying a silicone layer that extends beyond the length of the extension provides a visual reference of the original extension’s thickness.

Subsequently, excess silicone material hindering plate extraction is removed (Figure 15E), minimizing the necessary amount for extraction and subsequent material flow to these regions (Figure 15F). This process involves circular exposure of the alveolar ridge area while ensuring the surrounding silicone remains intact to prevent leakage during later stages. Any areas where excessive silicone is removed should be sealed with adhesive wax. If executed correctly, the prototype can be detached from the model using a plaster knife. Compressed air is then applied to the model and silicone assembly to remove any silicone remnants or dust (Figure 15G).

Then, adhesive wax is employed again to cover the cleft region in the silicone model setup, with special attention to undercut regions (commonly found around the alveolar ridge particularly in the posterior region) and deep incisions (Figure 15H). This blocking is crucial to ensure the safe detachment of the final manually manufactured TPP from the model base without causing breakage or damage. Afterwards, the setup is prepared by spraying 3D-printed model isolation material (Sheraseparat, HERA Werkstoff-Technologie GmbH & Co. KG, Lemförde, Germany) before applying the acrylate material. The isolation material is applied twice during the setup prior to subsequent material pouring. Between applications, excess material is removed using compressed air, after which the layer is allowed to air dry for one minute. To ensure accuracy and error-free production of the final part, the formation of puddles and contact with already sprayed areas must be avoided.

The base for the final TPP is crafted using cold-polymerizing polymethyl methacrylate (PMMA) (Orthocryl^®^, Dentaurum GmbH & Co. KG, Ispringen, Germany), composed of methyl methacrylate monomer (REF: 161-129-00) and polymethylmethacrylate powder (REF: 160-212-00). Application follows manufacturer’s instructions employing the “salt and pepper technique” [37], on the model and silicone extension. This entails alternating layers of powder and fluid to ensure controlled absorption without flooding the entire area (Figure 15I–K). A final layer of powder is added to maintain a dry top layer, reduce shrinkage, and enhance material accuracy [37]. Orthocryl colour concentrates (Dentaurum, blue REF: 161-129-00) are mixed with the monomer liquid to achieve coloration. The velopharyngeal extension is always coloured dark blue to provide optimal contrast during endoscopic evaluation. In contrast, the palatal base is coloured differently across plates to allow clear differentiation—typically red for the first plate, yellow for the second, and orange for the third. The appliance then undergoes polymerization in a pressure pot for a minimum of 20 min at 40 °C and 2.2 bar (Polymax 5, Dreve Dentamid, Unna, Germany), resulting in the raw TPP depicted in Figure 15I.

**Figure 16:**
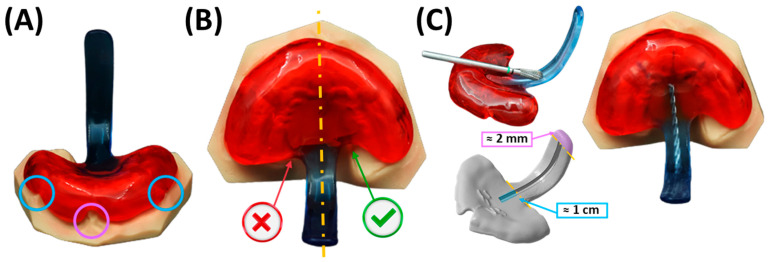
Manual processing of the TPP base palatal plate. (**A**) Spacing for the movement of the labial (purple) and buccal (blue) frenula. (**B**) Spacing needs to be provided on the posterior side of the palatal plate and close to the extension. Depiction of adequate (green) vs. inadequate (red) material removal on the posterior side of the base plate. (**C**) A groove is ground in the extension, from about 1 cm before the beginning of the extension and ending 2 mm before its tip, 1.5 mm in depth, where the safety wire will be implemented and polymerized.

Excess material (Figure 15L) is ground down, and the outer surface of the appliance is smoothed using a manual grinding handpiece with emery paper to achieve the final appliance shape (Figure 16). The thickness is maintained at 2–3 mm on the palatal base, ensuring the edges are not sharp. Material is removed around model structures representing the labial and buccal frenula (Figure 16A), ensuring space for proper functioning and movement of these structures. Similarly, increased posterior shortening of the plate is discouraged, as its length is crucial for maxillary retention. Finally, the upper extension width is narrowed, and the posterior edge of the plate is ground to a crescent shape (Figure 16B). These adjustments provide ample space for tissue movement, preventing OMLs.

The safety wire is integrated into the extension (Figure 16C), beginning approximately 1 cm from the start of the extension (posterior base palatal plate area) and ending approximately 1.5–2 mm before its tip. A groove of approximately 1.5 mm in depth is then milled into the extension to accommodate the placement of a pre-bent and pre-cut safety wire, which will be subsequently secured by polymerization. This facilitates the addition or removal of material in specific regions for necessary modifications during the course of therapy. The decision-making process is primarily informed by previous endoscopic findings and clinical experience, alongside clinical observations.

The safety wire, a stainless-steel rectangular reinforcement (cross-sectional area 1.8 × 0.8 mm^2^; REF: 312-106-00, Dentaurum GmbH & Co. KG), consists of twisted stainless-steel rods. The wire, pre-bent using pliers, is inserted seamlessly into the previously prepared groove without applying tension. To seal the groove, the plate side opposite the groove is covered with a covering mass (Gumex N, Dentaurum) to prevent monomer leakage and to enhance the strength and stability of the construction, particularly on the extension side, thereby ensuring that its original design remains unaltered. The groove containing the wire is subsequently sealed with Orthocryl.

During polymerization of the safety wire in the extension, particular care is taken to prevent bubble formation around the wire, as this could compromise the mechanical stability of the extension. To this end, Orthocryl is applied according to the manufacturer’s repair instructions, with the monomer placed in the groove prior to the polymer [37]. This technique ensures a secure bond with the existing material and minimizes the risk of bubble formation once the polymer powder is added. The application of liquid monomer helps maintain the material’s position, whereas powder particles are more prone to displacement, which could impair homogeneous polymerization within the groove. In this manner, the safety wire is firmly secured to the polymer extension, with layers of material placed beneath, above, and along both sides of the wire. Furthermore, the additional surface roughness provided by the twisted rods enhance wire–polymer integration.

Subsequently, the extraoral fixation bows are affixed to the TPP (Remaloy ^®^ straight wire, ø = 1.1 mm, 43 hard, strength = 1400–1600 N/mm^2^, REF: 528-110-00, Dentaurum) cut to a 6.5 cm length and pre-bent with a retention hook. These hooks are positioned on the palatal base plate; to determine their position, a second pair of palatal folds (canine position) is used as orientation points, with the wires being placed along this guidance. These areas can be previously marked on the model and then used as guidance for the grinding of a groove in the base palatal plate to accommodate the wires. Once again, the delicate position can be secured using covering mass to minimize part handling, preventing base plate distortion and wire movement during the polymerization process.

**Figure 17:**
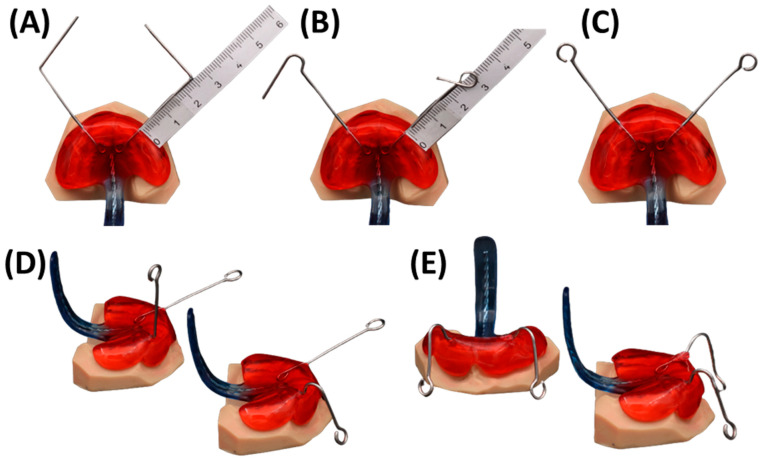
Bending of the extraoral fixation bows. (**A**) Distance to the start of the bending. (**B**) Final loop-like bending. (**C**) Final result of loop bending. (**D**) Lateral bending. (**E**) Frontal and side views showing finished extraoral fixation bows.

Once the bows are polymerized onto the base palatal plate, excess material is ground away to smooth the involved areas and ensure proper homogenization of the base plate. Subsequently, the kneading wax is removed, and the appliance undergoes additional finishing and polishing steps using a motorized polisher, as described above (Figure 10G,H). Afterwards, a calliper is used to control that the thickness of the plate is constant and not lower than 2.5 mm in any area, with the aim to reach a thickness of 2.5–3 mm. Variations are allowed in specific areas to accommodate clinical considerations (e.g., plate edges and highest alveolar ridge negatives or deepest plate areas). After the last polishing step, the extraoral fixation bows are bent (Figure 17). The wire is bent 2–3 cm after its exit from the base plate, for which hollow pliers are employed to create a loop in which the rubber rings will be subsequently placed. It is important that the loops are sufficiently closed to allow insertion of the rubber rings while preventing them from slipping out. Next, the wire exiting the groove is bent in an arc-like shape, first caudally and then following a U-shape cranially, achieving the final shape depicted in Figure 17E. The pre-bent shape should then be checked at the bedside to ensure that it spare the lips. Careful force application and consideration of plate handling is important to prevent damaging the material in the wire exit region from the base plate throughout the bending process.

Finally, once the appliance is completed, it must be thoroughly cleaned and disinfected. No residues of polishing or construction materials should remain. Cleaning may be performed manually using a toothbrush or similar instrument, or alternatively in an ultrasonic bath with a suitable cleaning solution. This process is then followed by steam-pressure cleaning, and the quality of the appliance is thoroughly inspected by the technician.

## 5. Quality Control, Potential Dangers and Side Effects

### 5.1. Appliance Quality Control Pre-Delivery

Before the plate is delivered to the patient, a thorough examination must be performed by the orthodontist to ensure that the medical appliance is free of any polymerization bubbles or micro cracks that could compromise its mechanical stability and safety [24]. If damaged regions are identified, they should be repaired or replaced according to the manufacturer’s instructions. This typically involves grinding the affected areas, followed by subsequent polymerization, and may require repeating some of the steps outlined above. Although such damage is highly unlikely given the prior quality control conducted in the dental laboratory before delivery, these measures are essential to ensure the quality and safety of the medical appliance provided to the patient.

Particular attention must be given to the velopharyngeal extension. It must be confirmed that it is rigid and shows no flexibility. Insufficient thickness of the extension, i.e., less than 2.5–3 mm, would make the employed combination of Orthocryl and safety wire unstable. The safety wire must be precisely centred within the palatal plate, embedded without surrounding bubbles, and securely polymerized to the extension. The extension itself must be centred and fully supported by the wire, with the exception of the tip of the extension. Here, approximately 2 mm of material is deliberately left to allow for adjustment and shortening of the extension during the fitting procedure bedside.

Furthermore, the base plate must be inspected to ensure a secure, rotation-free fit on the printed maxillary model. All sharp edges must be removed, and thorough polishing must be performed. The extraoral fixation bows must be centrally placed on the base plate, and the bow ends must be rounded and smoothly polished to avoid potential injuries.

During the fitting and evaluation process, several safety and functional aspects must be ensured. The large bend of the extraoral fixation bows emerging from the base plate must be carefully shaped to avoid excessive force, which could cause fractures in the polymer material at the wire exit. Moreover, the wires’ curvature should be adjusted to accommodate the patient’s lips comfortably. Regarding general safety parameters, the minimal thickness of the plate must be maintained, particularly in the velopharyngeal extension. While adjustments to the extension’s length and width are permissible to optimize fit and function, any modifications must maintain structural stability to avoid compromising patient safety. Throughout the fitting process, the patient must be closely monitored. The only instance in which the safety wire may be absent is during endoscopic evaluation. At all other times, the safety wire must remain in place.

### 5.2. Final Fitting Appliance Evaluation, TPP Insertion, Adjustment and Initial Handling Protocol

Prior to insertion of the TPP, the responsible clinician must ensure that the oral mucosa is intact and free of food or milk residues. This is typically assessed using a flashlight and wooden spatula, if necessary. The oral cavity is then gently cleansed using a gauze swab soaked in dexpanthenol solution.

#### 5.2.1. Adjustment of the Extraoral Fixation Bows and Frenula Sparing of the Base Palatal Plate

During the initial application, the extraoral fixation bows require manual adjustment by the clinician, ideally a trained orthodontist. For this purpose, the use of a standard wire-bending pliers and a three-point bending pliers is recommended. The TPP should initially be inserted without adhesive to evaluate the bow positioning in relation to the upper lip. The fixation bows must not exert pressure or create notches on the lip, as this may result in OMLs. If necessary, repeated insertion and removal may be required to optimize the bow alignment extraorally. Additionally, the bows should be adjusted to a width that permits bottle feeding without causing obstruction, ensuring that the device does not interfere with the feeding process. This adjustment should not be too extreme at the beginning but rather modified progressively to accommodate the infant’s needs. Otherwise, there is a risk of injuring the corners of the lips.

Once the bows are correctly positioned without interfering with physiological lip movement, the fit of the palatal base plate must be evaluated. Particular attention should be paid to the frontal and lateral labial frenula to ensure that the base plate provides sufficient clearance and does not cause mucosal irritation. If contact with these structures occurs, the excess material should be removed and polished at bedside using a manual grinder.

#### 5.2.2. Final Insertion

Once the appliance has been adjusted and fit-tested, adhesive cream (Procter & Gamble, Schwalbach, Germany) is applied to the palatal base plate. Application should be limited to the anterior region near the labial frenula and the lateral margins, avoiding the central or cleft area to prevent adhesive from entering the cleft or nasal cavity. Approximately three pea-sized drops are applied and spread across the lateral and anterior regions of the arch, carefully avoiding the cleft area. The adhesive enhances retention, particularly during swallowing, when tongue pressure increases.

**Figure 18:**
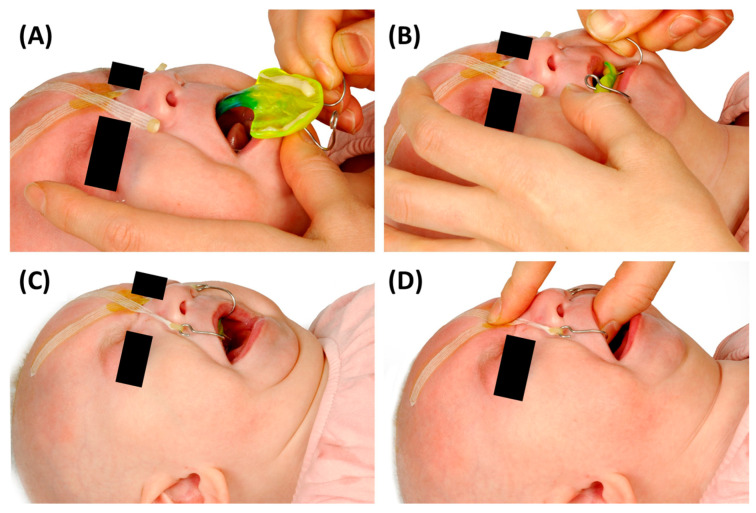
Insertion and fixation of the TPP in an infant with RS in supine position by the caregiver. (**A**) Initiation of insertion with head stabilization. (**B**) Placement of the velopharyngeal extension on the tongue and palatal base plate on the maxilla, avoiding posterior displacement of the tongue. (**C**) Correct intraoral positioning after securing the extraoral wires with orthodontic elastics. (**D**) Final stabilization by holding the plate in position for 20 s to several minutes to ensure adhesive setting.

For initial fittings, the insertion should be performed by two trained clinicians. One person stabilizes the infant’s head with both hands from above, while the other inserts the TPP (Figure 18). Insertion proceeds in three steps: placement of the velopharyngeal extension on the posterior tongue, guidance of the extension into the upper airway, and positioning of the palatal base plate on the maxilla.

Mild pressure may be applied to guide the tongue anteriorly with the device. Caution must be taken to avoid posterior displacement of the tongue, which may compromise the airway. A correct insertion results in an anterior, physiological tongue position, as illustrated in Figure 18. If this is not achieved, the TPP should be immediately removed and reinserted. The base plate must be checked for fit, ensuring it is stable, secure, and cannot rotate or wobble on the maxilla.

#### 5.2.3. Fixation Technique

To stabilize the appliance, two adhesive tapes (Steri-Strip, 3M Health Care, MN, USA), each pre-fitted with an orthodontic elastic band (intraoral, non-latex, medium pull, 1.3 N, 1/8 inch; Dentaurum, Ispringen, Germany), are threaded through the extraoral fixation bows and crossed over the forehead under adequate traction. Prior to application, an adhesive enhancer such as Benzoin tincture 90% 1:5 (Caelo, Hilden, Germany) is applied to the forehead using a sterile swab and allowed to dry completely to optimize adhesion.

**Figure 19:**
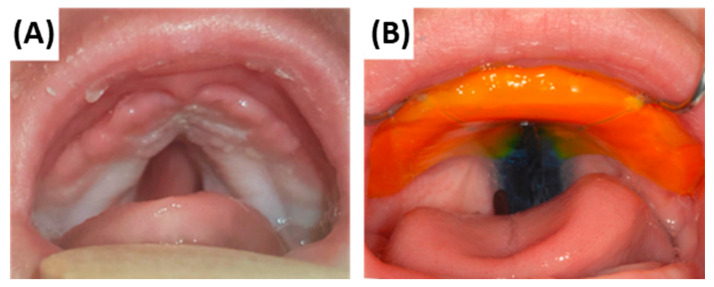
Intraoral views of a RS patient with a cleft palate. (**A**) Baseline intraoral situation without the TPP. (**B**) Correctly inserted TPP with orange palatal base plate, extraoral fixation bows, and blue velopharyngeal extension with safety wire. The extension occupies the cleft space without impinging on the soft palate muscles, allowing physiological swallowing. With the TPP device, the tongue is maintained in a ventral and physiological position.

Insufficient traction may compromise the stability of the velopharyngeal extension, while excessive tension can cause discomfort, skin irritation, or pronounced skin folds. However, minor skin folding is generally considered acceptable, and sometimes even necessary. Once positioned, the appliance should be held in place for between 20 s and several minutes to ensure that the adhesive sets fully and secures the TPP in its correct position (Figure 19).

In patients with sensitive skin, irritation from the adhesive enhancer or tape may occur, particularly on the forehead, where redness can develop at the contact sites. In such cases, repositioning the tapes slightly more laterally or medially than usual is recommended. Additionally, the width of the adhesive tapes can be adjusted: narrower strips may reduce skin irritation by minimizing contact surface, while wider tapes may provide better adhesion during warm weather conditions or in infants prone to sweating.

#### 5.2.4. Position of the Velopharyngeal Extension

Subsequently, the position of the velopharyngeal extension is verified upon the first final TPP insertion (Figure 19). The extension should occupy the cleft space adequately while allowing unimpeded movement during swallowing. During the first application, the infant should be closely monitored by the orthodontist to assess tolerance. Persistent gagging or vomiting during this period may indicate an excessively long extension, which should then be shortened. Conversely, limited tongue mobility or impaired feeding suggests that the extension is positioned too anteriorly. The primary goal is to reduce or eliminate stridor immediately after insertion. Following this initial assessment by the orthodontist, trained nurses will continue to monitor the patient’s response to the appliance. Most infants tolerate the device after an acclimatization period ranging from several minutes to a few hours. However, feeding with the TPP requires adaptation and should be supported by guided training over the course of several days, always starting with feeder-assisted feeding [14].

#### 5.2.5. Daily Maintenance and Reapplication

The TPP must be removed once daily for inspection and hygiene. Unlike other areas of the appliance, the base plate is not highly polished; the grooves corresponding to the palatal structures further increase the perceived roughness, while avoiding any sharp edges that could cause injury. This texture is intentional to promote retention of the adhesive paste [25]. However, this also facilitates bacterial proliferation, making daily removal and cleaning essential [38,39]. If the fixation tapes remain effective, daily replacement of the tapes is not mandatory. During removal, one hand stabilizes the adhesive strips on the forehead and supports the plate inside the mouth with a finger, while the other hand detaches the elastic bands from the fixation bows. After removal, both the appliance and the oral cavity must be thoroughly cleaned. The appliance can be cleaned by gentle brushing with non-abrasive toothpaste, by using cleaners or disinfectants formulated for oral acrylic appliances, or, if available, by ultrasonic cleaning [38]. In all cases, harsh chemicals, high temperatures, and excessive mechanical abrasion must be avoided. Once cleaned, the TPP may be reinserted, and the fixation process repeated. Additional practical aspects of daily care, including nursing support and handling techniques, have been described in detail elsewhere [14].

It must be emphasized that thorough training of caregivers is essential to ensure the safe use and proper maintenance of the appliance. Adequate instruction helps prevent handling errors that could compromise the stability or function of the TPP. For example, disinfection methods such as boiling—although sometimes attempted—are not recommended, as they fall outside the material specifications provided by the manufacturer and may damage the appliance. Daily inspection of the appliance by parents must be encouraged to ensure it remains in good condition—checking for breakage, sharp edges, or unwanted shifting of the extraoral fixation bows. Additionally, all parents receive training in cardiopulmonary resuscitation prior to hospital discharge.

### 5.3. Follow-Up Controls and Addressing Potential Side Effects

Patients are discharged after a favourable sleep study (OAI < 3/h), adequate feeding behaviour, and weight gain. Equally important is caregiver training, which ensures correct device use, maintenance, and regular oral mucosal inspection, preventing misalignment and enabling early identification of irregularities. Caregiver confidence with appliance handling is a key criterion for discharge. Patients leave with a portable oxygen saturation device, and regular outpatient follow-ups allow early detection of OMLs and progression of vestibular indentations caused by the TPP, facilitating timely appliance adjustments to accommodate growth and enhance comfort.

#### 5.3.1. Oral Mucosal Lesions (OMLs) or Mucosal Ulcerations (MUs)

**Figure 20:**
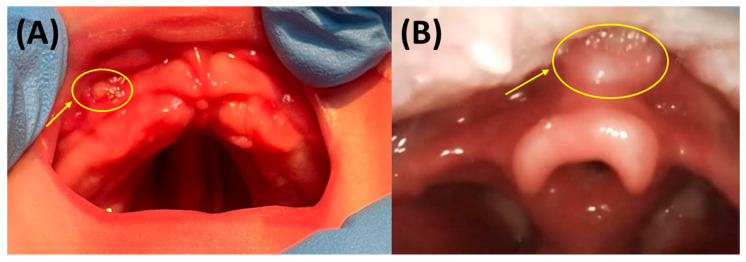
Different types of MUs (highlighted in yellow). (**A**) MU on the left vestibule of a patient treated with the TPP, alongside an indentation caused by an insufficient palatal plate vestibular wall and palatal plate outgrown by the patient. (**B**) Endoscopic view of a MU located in the vallecula, between the root of the tongue and the epiglottis.

Regular appliance checks and mucosal inspections during daily follow-ups are essential for early detection and management of complications. During these assessments, device fit and treatment progress are routinely assessed, and oral mucosal lesions (OMLs)—such as mucosal ulcerations (MUs), often called “pressure marks”—are documented. These lesions typically appear as oval, white spots with red borders, about the size of a lentil (Figure 20A) [32,40]. They must be addressed before continuing treatment, usually by grinding and polishing of the appliance at the bedside to relieve pressure and promote healing [32]. These adjustments must be performed with great care to avoid excessive grinding of the vestibular wall, which may compromise its thickness and length, or thinning of the basal or middle plate regions, both of which can reduce accuracy and weaken the TPP’s retention on the maxilla. Such alterations risk mispositioning of the base plate and may lead to new OMLs. Ensuring an adequate vestibular wall, careful grinding, and sufficient palatal extension is essential not only for reliable TPP retention but also for homogeneous load distribution and prevention of further mucosal injuries. In some cases, a new intraoral scan and plate fabrication are necessary. Due to the forces exerted by TPP devices during physiological movement and feeding, mucosal strain and ulcerations are more common, affecting especially the maxilla. One study found the vestibule was the most frequent site (31%), followed by the lip frenulum (14%) and tuber region (11%) [40].

In rare cases, an OML may develop in the vallecula—the space between the tongue root and epiglottis (Figure 20B)—usually caused by an excessively long velopharyngeal extension. Clinical signs include newly developed swallowing difficulties, fever, and restlessness [7]. An endoscopic examination is required to confirm the presence of a vallecular OML. In such situations, the deliberately retained 2 mm of material at the tip of the extension allows for bedside shortening to relieve pressure. In some cases, inadequate retention of the palatal plate—due to factors such as insufficient vestibular walls of the palatal plate or flat alveolar ridges of the patient—may lead to displacement and subsequent pressure on this region. Notably, such lesions typically occur only during fitting while the patient is still an inpatient at our centre and are exceedingly rare after discharge [40]. In some cases, discrepancies in the perceived length or positioning of the extension during endoscopy have been observed. These variations may be related to differences in patient positioning, particularly head placement, which can influence the apparent extension length. This highlights the importance of standardized positioning during endoscopic procedures to ensure consistent and accurate assessments.

Emphasis should be placed on the accurate design and production of the TPP to reduce the probability of OMLs. The first days following insertion are crucial for monitoring, adjustment, and ensuring that the patient adapts successfully. While patients gradually accommodate to the device, this process depends heavily on optimal appliance configuration. Errors in data acquisition—whether via IOS or conventional alginate impressions—can lead to ill-fitting plates and mucosal damage. A well-adapted extension and proper swallowing mechanics contribute to the stabilization of the appliance, significantly reducing movement and, therefore, the risk of OMLs.

Additionally, the authors hypothesize that several factors—including patient adaptation to the appliance, mucosal response, and feeding strategies—play a key role in the development or prevention of OMLs in RS patients. One important consideration is the distinct nature of the oral mucosa in newborns compared to older infants. For example, we have observed that in infants with unilateral or bilateral cleft lip and palate, tongue-induced forces can occasionally produce an OML at the vomer already in utero, which only becomes visible at birth [40] and typically resolves spontaneously if the area is left exposed. In patients with cleft and/or lip palate (CL/P) and RS undergoing palatal plate therapy, such lesions healed within a few days when space was provided or a short therapy pause was implemented [7,32,40]. Weismann et al. reported an average healing time of five days [40], which may be shortened by temporarily pausing appliance use. This fast healing may be attributed to the oral mucosa’s accelerated cell turnover, likely an adaptation to its high functional demands [40]. Such regenerative capacity supports rapid healing and adjustment to OMLs from the oral appliance.

We hypothesize that the mucosa may be more delicate in the first days of life and requires a gradual adaptation to functional stresses. Since RS patients using a TPP experience greater oral demands than healthy infants, a gradual increase in feeding-related strain is recommended to minimize mucosal complications associated with the appliance. Our feeding protocol thus follows a structured progression, starting with feeder feeding (Finger Feeder, Medela, Baar, Switzerland) to promote anterior tongue movement. Finger feeding must be avoided, and the use of pacifiers should be minimized as much as possible. During active sucking, small amounts of milk are administered in synchrony with each swallow. After TPP placement, feeding continues with a feeder but without finger assistance to minimize pressure on the palatal base [7,14]. Bottle feeding is introduced only after OMLs have healed, the TPP fits properly, and the tongue can reach the teat. At this stage, bottles with variable milk flow, such as Playtex Baby™ Drop-Ins^®^ (Shelton, CT, USA), are recommended. The timing for starting bottle feeding is highly individual and typically takes 1–2 weeks before intermittent bottle feeding can be gradually introduced for training. Children are often discharged after 2–3 weeks of feeder feeding with increasing bottle use. Complete transition to bottle feeding is not a discharge criterion in our clinic. Further details on this matter are available elsewhere [7,14].

#### 5.3.2. Mucosal Indentations or So-Called “Dents”

**Figure 21:**
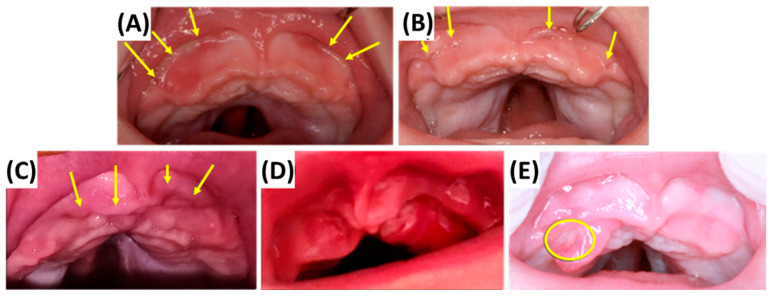
Oral mucosal indentations in patients with RS treated with TPP. (**A**,**B**) Patients with cleft palate showing circular indentations without signs of inflammation. (**C**) Indentation in the anterior maxilla caused by a malpositioned TPP, displaced posteriorly by the tongue. (**D**) Deep indentation in the anterior maxilla with two pronounced MUs indicating an inflammatory stage. (**E**) Indentation with mucosal fibrosis and a MU on the right side of the maxilla.

In patients with RS, it is essential to distinguish between OMLs and indentations, commonly referred to as “dents.” The latter typically have a cascade-like appearance and are primarily located in the mucosal vestibular fold and along the alveolar ridge, often extending towards the medial labial frenulum. Initially only palpable, they later become visible upon clinical inspection (Figure 21A,B) and are due to counterbalancing forces exerted by the appliance—specifically, the mechanical translation of forces applied at the base of the tongue.

Such dents are considered physiologically acceptable and occur in nearly all patients, resulting from the combination of the above dynamics, natural maxillary growth, and the progressive mismatch between the appliance and the growing anatomy. Consequently, a new TPP is often fabricated, which is explained in a subsequent chapter.

Figure 21C illustrates a clinical case in which the TPP was malpositioned over a period of time, resting on the alveolar ridge or even further posteriorly on the maxilla. In such cases, the appliance no longer fits correctly, either due to misplacement or physiologic growth. If growth is the cause, the extension is often too short in the sagittal dimension, signalling the need for a new device. Pronounced dents can also be the result of improper extension configuration, such as excessive pressure on the tongue or an excessively sagittal anterior position. Without timely intervention, these pressure-related dents can deepen into the mucosa, progressing into dark red, painful MUs as early signs of inflammation (Figure 21D), and potentially worsening further with mucosal fibrosis (Figure 21E). Mucosal fibrosis may also occur when the palatal base plate lacks adequate precision or surface accuracy. These outcomes are considered unacceptable and necessitate urgent fabrication of a new TPP.

It is important to emphasize that while minor dents are an expected and acceptable finding during treatment, they must be closely monitored. If there are any major dents, signs of inflammation or mucosal breakdown (e.g., MUs or fibrosis), continued use of the current appliance is contraindicated. Before manufacturing a new device, however, a short pause in TPP therapy is recommended to allow for mucosal healing. After the healing is complete and no dent can be observed, a new intraoral scan is performed for a new appliance.

During such interruptions in TPP treatment, respiratory monitoring becomes critical. Patients should be equipped with a pulse oximeter to detect potential airway compromise at home. Additionally, both the patient and caregivers, must remain in close proximity to the hospital during this period for immediate access to medical support if needed. At our centre, patients stay in family rooms that have been especially adapted for such needs.

### 5.4. Checklist for the TPP Protocol

Given the numerous critical elements required for the successful implementation of TPP therapy and the detailed nature of the protocol, the checklist shown in Table 1 provides a concise summary and may serve as a practical guideline for other clinical centres.

## 6. Reasoning for the Current TPP Approach and Workflow in Our Centre

**Figure 22:**
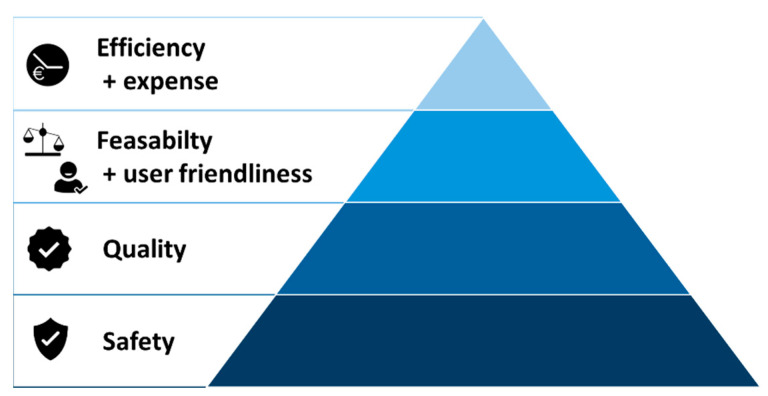
Key factors for implementing a new treatment or workflow for a medical appliance (adapted based on [32]).

Any new clinical approach or technology must follow the decision pyramid in Figure 22, prioritizing patient safety above all. Additional factors, in order of relevance, are quality, feasibility, and user-friendliness, then efficiency and cost. These criteria are essential to assess before adopting any method in a cleft centre [32]. Since the TPP offers a less invasive alternative to surgery with comparable functional outcomes, it should be preferred whenever appropriate—provided it is manufactured and applied under strict safety standards. The authors stress the critical importance of ensuring the safety of personalized medical appliances, especially in vulnerable patients like newborns. Before implementing new workflows or materials, safety and risks must be evaluated, starting with in vitro assessments. Additionally, the current lack of standardized safety controls in personalized appliance manufacturing must be addressed; facilities introducing new materials or technologies should perform thorough pre-evaluations across all safety domains.

The presented semi-digital workflow for TPP manufacturing combines digital design and AM of prototypes with conventional fabrication of the final appliance. This approach is particularly recommended because IOS offers a safer and more reliable method of data acquisition for patients with RS, avoiding the risks associated with alginate impressions [18,20]. Beyond safety, the semi-digital workflow enhances quality, efficiency, and reduces the need for repeated endoscopy appointments. It also facilitates the generation of comprehensive digital patient records via IOS and CAD, enabling streamlined data exchange between centres, remote assistance, and training [32]. Moreover, standardized digital data supports future multicentre, prospective randomized studies aimed at improving outcomes and patient care. For centres seeking to reduce costs, the digital design and AM of prototypes can be omitted: IOS can still be used to ensure safety, with printed digital models and conventionally fabricated prototypes for endoscopy (see Section 8 on subsequent TPPs).

### 6.1. Reasoning for the Presented Prototyping Approach

The TPP consists of a patient-specific palatal plate based on the patient’s IOS-derived maxillary data, along with a velopharyngeal extension that is initially standardized during the prototyping stage. This decision is multifactorial, as fitting the extension is challenging due to the lack of information about pharyngeal shape and length [24]. Although studies have proposed advanced imaging techniques such as magnetic resonance imaging (MRI) or computed tomography (CT) [26], which could provide pharyngeal geometry, we consider these methods unnecessary and thus unethical [8]. In some cases, transporting patients—especially those connected to medical devices—is difficult or unfeasible. CT imaging entails radiation exposure, elevating risks of cancer, brain tumours [41,42,43], and lens damage [44,45], among other concerns, particularly in young patients. Moreover, while imaging can offer insights into factors such as extension length and initial sagittal positioning, it can only provide a “snapshot” of the anatomical structures for the digital design. The images are typically captured in a specific posture (e.g., supine), which does not reflect the dynamic physiological conditions [8]. It fails to account for functional dynamics such as swallowing and breathing. Because the TPP must interact with tongue and pharyngeal movements, awake endoscopy remains essential to assess extension positioning in relation to muscle contractions and epiglottic movement [8]. Furthermore, possible anesthesia could distort these conditions by relaxing the pharyngeal muscles. Incorporating additional imaging steps that offer limited clinical value would unnecessarily increase the burden on healthcare facilities, patients, and caregivers [8]. This approach would also contradict the decision pyramid for workflow implementation at different levels (Figure 22). Therefore, the authors advise against designing these appliances based solely on imaging techniques, emphasizing instead the essential role of awake endoscopy during the prototyping phase.

A standardized prototyping approach is preferred to ensure consistent quality and mechanical properties across all patients. Standardizing the extension’s thickness, curvature, and width is essential for safety and predictable mechanical behaviour of the prototype during endoscopy, as forces from the patient’s stomatognathic system vary and increase with the extent of retrognathia and glossoptosis. A standardized extension ensures uniform treatment quality across patients. Furthermore, the uniform extension thickness serves as the basis for the final appliance. As a result, each prototype provides a reliable reference for a stable TPP configuration and should only be modified with caution to preserve its structural integrity and functional effectiveness.

A critical aspect of prototyping is the position of the velopharyngeal extension along the sagittal plane—specifically, determining whether it requires a more anterior or posterior position. To address this, providing multiple prototypes with varying sagittal positions is recommended. DLP machines enable the simultaneous fabrication of these prototypes, significantly reducing production time and making this approach feasible within a clinically relevant timeframe. As a result, multiple sagittal positions can be assessed during a single endoscopic session, thereby minimizing the burden on the patient, caregivers, and clinical staff. Based on clinical experience, the provision of three prototypes, each with 2 mm variation in the sagittal direction, has proven sufficient. Producing more than three prototypes offers limited additional value, as the final appliance will still be manually fabricated and can accommodate further positional refinements if necessary. Moreover, exceeding this number of prototypes may increase patient discomfort during the evaluation and lead to unnecessary manual post-processing efforts.

### 6.2. Reasons for a Conventional Manufacturing Approach for the Final TPP Appliance

**Figure 23:**
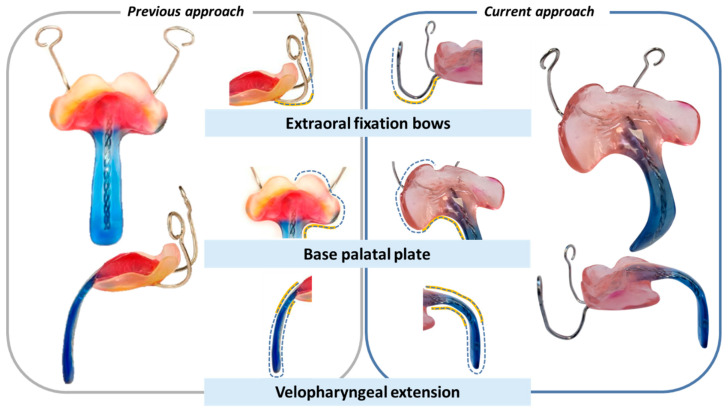
TPP changes and optimizations from previous to current approach.

The final appliance, fabricated using conventional methods, represents an optimized version of the earlier TPP design shown in Figure 23. These refinements were gradually introduced by the interdisciplinary team to address specific clinical challenges. A visual comparison is provided between an early TPP from initial studies and a representative appliance produced using the current workflow. While a direct comparison is limited—due to differences in patients and dental technicians—several key improvements are noteworthy. In the earlier design, the extraoral fixation exited the base plate without pre-bending, resulting in a straighter curve. In contrast, the current version features a rounded transition a few millimetres beyond the plate, reducing pressure on the patient’s lip. Posteriorly, the base plate is intentionally shortened to preserve soft tissue mobility, prevent OMLs in the cleft region, and facilitate feeding. Additionally, the extension’s inclination has been modified to a more curved form, with the distal end now aligned parallel to the tongue base, ensuring better force distribution and improved functional outcomes.

The reason to maintain a conventional manufacturing approach for the definitive plate is mainly based on our safety-first principle, i.e., the use of a safety wire that secures the appliance’s extension against breakage [24], which is particularly important in infants treated at home, e.g., without constant clinical supervision. This context underscores the necessity of thoroughly validated and fail-safe designs in the production of the final therapeutic appliance. From the experience gathered in our facility, appliances may suffer breaks, cracks, or defects due to improper use—such as accidentally stepping on it, dropping it, or inadequate maintenance and cleaning practices (e.g., disinfecting with high temperatures compromising material stability). We caution against caregiver negligence in response to fissures or microcracks in the device that may result in fragmentation and aspiration of parts by the child, potentially leading to asphyxia, given the limited coughing ability of young patients. Although such scenarios have not been reported in our facility, authors stress the responsibility of facilities to anticipate various risk scenarios and assess the appliance’s response, along with providing proper caregiver information and training. To mitigate this risk, the appliance must include a safety wire with sufficient retention to prevent detached parts from sliding downward. In the presented approach, this is achieved using a twisted wire, which provides mechanical security in the event of breakage and significantly enhances overall safety.

**Figure 24:**
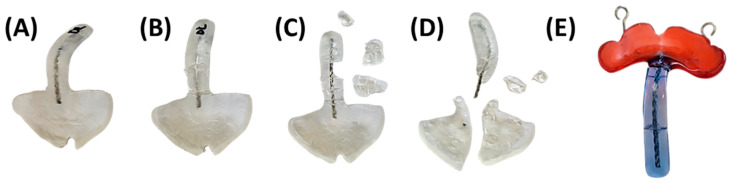
Potential breaks, cracks and defects simulated on TPPs. (**A**–**D**) Show the potential fracture behaviour of a TPP under applied forces on a study made on AM materials (recreated based on a previous study [24]), from best scenario (**A**) where not breakage occurs no matter the applied load or a breakage occurs (**B**)), where the safety wire avoid fracture piece spread; to worse-case scenarios with loosening of particles (**C**) or complete detachment of the extension from base plate (**D**), which could potentially lead to material aspiration by the patient. (**E**) Simulated breakage and unintentional bending of bows caused by stepping on a conventionally manufactured TPP, showcasing the same behaviour as case (**B**).

In a previous study, integrating a safety wire into TPPs manufactured via AM and SM proved cumbersome [24] (Figure 24A). The fracture strength required to pose clinical risk must be assessed alongside the fracture behaviour of appliances made from different materials and technologies (Figure 24B–E). Among digitally manufactured options, only high-performance polyetheretherketone (PEEK), produced via SM, demonstrated sufficient mechanical integrity without a safety wire. However, its high cost and complex workflow integration (as outlined in Figure 22) make it impractical for routine clinical use. While some AM materials exhibited high fracture strength, testing was limited to a standard extension shape—fixed in width, thickness, and curvature. Moreover, the fracture behaviour of other medically approved, commercially available materials—both with and without integrated safety wire—was inferior to conventionally manufactured appliances [24]. Many showed poor mechanical reliability under stress, raising safety concerns. Additionally, most medically approved materials with high flexural strength were transparent, with only a few available in coloured variants suitable for clinical use. It is crucial to account for forces not only during intraoral use but also when the appliance is handled outside the patient´s mouth. Given these risks, the authors strongly discourage using TPPs without a safety wire in RS patients and caution against using materials lacking documented safety margins, as supported by prior findings [24].

The production of the final TPP using AM and SM may become feasible in the near future. With rapid advances in materials and technology, broader availability of medically approved, colourful, and high–flexural-strength materials can be expected. Increased adoption of digital tools—such as IOS, AM, and SM devices—will further support the transition toward fully digital TPP workflows.

As these medical appliances are non-standardized, personalized medical devices, there are currently no established design or manufacturing quality controls beyond basic medical material approval. We wish to stress the importance of ensuring these devices are safe. Before implementing any new workflow involving personalized appliances, cleft centres must prioritize patient safety and proactively address potential risks. To the authors’ knowledge, none of the published literature from other centres provides quality control protocols or defines minimum safety standards for these appliances.

## 7. Other Workflow Approaches

While various centres worldwide have introduced alternative TPP workflows, relatively few have been published. Most acknowledge the TPP protocol as a precedent [26,46,47,48,49,50,51,52,53,54,55], while others refer only to Pielou’s 1967 description of a slightly tipped palatal plate, without mentioning any prior use of a velopharyngeal extension [51,53]. Although the concept of a velopharyngeal extension originates from Pielou, the TPP differs significantly: its longer extension reaches just above the epiglottis, advancing the tongue root, elevating the epiglottis, and opening the larynx to prevent obstruction. Unlike Pielou’s design, TPP fitting requires awake endoscopy due to the effects of sedation on airway patency and the limited laryngeal space [56]. The Tübingen Palatal Plate (TPP) was first introduced as a term in the scientific literature as the German term “Tübinger Gaumenplatte” by Bodmann et al. in 2003 in Tübingen. The same team later adapted the terminology to English, referring to it as the Preepiglottic Baton Plate (PEBP) in 2007 [57], before reverting to the term TPP. While PEBP and TPP remain the most widely used terms in the literature, the designation Orthodontic Airway Plate (OAP) has only recently been introduced by another group [53,54], although it describes the same underlying principle already established earlier.

Almost all documented approaches start with a prototype followed by a final appliance, both are mostly produced using conventional dental manufacturing methods, with varying shapes of extraoral fixation bows and velopharyngeal extension (Table 2). Most lack comprehensive details on materials, manufacturing processes, and integration into interdisciplinary workflows. One proof-of-concept exists for a fully 3D-printed appliance, including extraoral fixation bows [26]. However, this is not yet feasible in daily clinical practice, primarily due to safety concerns over the lack of a safety wire and the impracticality of extraoral facial scanning and manufacturing of fixation bows. Metal bows remain easier to attach and adjust during treatment, whereas rigid printed ones would require reprinting each time an adjustment is needed.

Some authors have proposed dynamic palatal base plate extensions, usually consisting of single rounded orthodontic rods that can be bent during the prototyping phase [48,49,51,52,53,55]. While intended to simplify prototype fitting, these setups are often retained in the final TPP appliance. Although seemingly practical, this approach carries significant drawbacks. The most common dynamic prototyping approaches [48,49,51,52,53,55] typically allow a single bend in the upper third of the velopharyngeal extension, near the palatal plate. This segment can usually be manually adjusted, for example, with pliers, and is made of wires, thermoplastic materials, or similar components. Unlike the rigid polymer design described in this manuscript, these setups lack structural stability to maintain the intended configuration. 

Firstly, authors emphasize that during the prototyping stage and endoscopic evaluation, effective airway opening does not depend on achieving a perfect extension angle. This is determined by how the shape of the extension governs force distribution. The extension does not form a true ‘angle’, but should be understood as a curvature or spline, particularly in the upper cleft segment (‘cleft extension’) that connects the base plate to the extension. This part does not affect airway opening, though it may influence feeding depending on cleft morphology and design. The effective extension—extending from the occlusal surface downward—is the critical functional component. Airway opening depends on correctly positioning the functional portion, especially the last two-thirds, along the sagittal plane (Figure 1D and Figure 13) [8]. This ensures broad, even contact with the tongue and distributes forces homogeneously. In contrast, employing dynamic setups only considering the angle rather than the sagittal position risks the creation of a pronounced angle may fail to open the upper section effectively, concentrate forces locally, and increase the risk of OMLs on the tongue, causing pain, discomfort, and potential feeding or sleep issues. OMLs in the pharyngeal region—detectable only endoscopically after prolonged device use (Figure 20)—represent an even greater concern. The lower third or tip of the extension, usually oriented anteriorly to avoid the epiglottis, follows anatomical shape without generating additional force; this is the only minimal ‘angle,’ typically just a few degrees. In summary, sagittal placement is decisive: shifting the extension anteriorly or posteriorly alters force distribution and airway opening by positional change, not by creating a meaningful angle.

While most dynamic systems employ two rounded rods [46,49,50,51,52], one approach uses a single rod [55]. In this method, a dynamic setup is applied during prototyping, followed by a static configuration with two rods for safety, similar to the approach proposed here. However, during endoscopy and the initial fitting phase, only a single wire connects the palatal plate to the extension. Such a prototype is highly unstable and may not provide sufficient airway opening. We hypothesize that this design is particularly risky: not only is it more prone to breakage, but when translated to the final TPP, it may allow not just positional shifts but also unwanted or intentional rotation of the extension. 

Apart from setups using one or two rounded rods, one approach employs rods bent into an undulating “omega” shaped to mimic an extension, without any polymer support on the extension [48]. This setup shares the same drawbacks as the previous approaches. Although any force-adjustable dynamic setup carries some risk of displacement, this risk is most pronounced in single-wire systems. Importantly, no study proposing wire-bending dynamics has examined potential elongation or displacement under tongue forces, raising further concerns about stability, unintended movement of the plate–extension assembly, and misinterpretation of endoscopic results, both during inpatient fitting and after hospital discharge.

One approach proposes using screws to adjust the length of the velopharyngeal extension [49]. However, cleaning and disinfection are difficult [39], and contact with pharyngeal mucosa and food increases irritation risk, as these screws were never designed for pharyngeal use. Their thickness further limits applicability, since no commercially available screw is thinner than the recommended safety wire, preventing low-profile extensions. As dynamic elements, screws can be unintentionally altered after discharge, while the wire compromises both force distribution and stability. While there is no peer-reviewed evidence directly linking a screw’s small retention area within polymer to increased breakage rate, mechanical principles and appliance component fatigue suggest that stress concentration at such embedded interfaces could plausibly elevate failure risk. Although formal testing is needed, screws clearly add instability, infection risk, and failure potential, without offering advantages beyond simpler methods. Moreover, length adjustment is the least demanding step of the TPP workflow and can be readily achieved by bedside grinding of the polymer.

Another approach proposes using thermoplastic materials during the prototyping phase of the TPP [54]. However, thermoplastics lack rigidity and may not provide sufficient stability for a treatment that requires a stable extension to counteract tongue obstruction in the pharyngeal area. Increasing thickness could improve stability, but this is undesirable when the velopharyngeal extension should remain relatively thin to be comfortable for the patient. The authors neither specify the thermoplastic material used nor address the bending behaviour of the extension; it is reasonable to assume that thermoplastics alone cannot adequately bear functional forces. While they propose a polymer–thermoplastic–polymer layered final TPP to improve stability, it can still offer limited stability at the cost of greatly increased thickness. Additionally, the safety of the thermoplastic–polymer bond remains unexamined and not addressed in this methodology. Consequently, it is safe to assume this configuration does not meet the necessary safety or load-bearing standards. Furthermore, using thermoplastic during endoscopy may mislead clinicians regarding actual force application, and a direct transfer of such a dynamic prototype to the final TPP appears highly improbable.

Finally, while most approaches incorporate two rounded rods for safety, there is one approach relying solely on a static polymer extension without any safety wire [53]. Commercial rounded wires are generally thicker than the safety wire proposed in this study; when braided, they may achieve comparable performance to the flat braided wire described here, as the polymer interlocks between strands and improves retention. In contrast, single rounded wires can be easily dislodged. Meanwhile, the authors emphasize the importance of including a safety wire, as omitting it presents a significant risk, as previously discussed. Ensuring sufficient safety is critical in newborns, who—unlike adults—cannot expel fragments if a fracture occurs. Since implementing the polymer–safety wire combination at our centre, no breakages have been reported intraorally. Nevertheless, it is essential to consider forces both during intraoral use and during handling outside the patient. Studies using polymer extensions alone provide no validation of safety margins or force resistance without a wire [53]. Given the vulnerability of the patient population, cleft centres must ensure that intraoral breakage cannot occur; even if unnoticed cracks develop outside the patient, the safety wire must prevent fragments from being swallowed (Figure 24B). While no emergency scenarios from appliance safety failures have been reported, the absence of foundational and in vitro studies on extension-based appliances is concerning. With the exception of Thurzo et al., who proposed minimal mechanical standards in a proof-of-concept study [26], none of the published approaches include systematic quality or safety testing [48,49,51,52,53,54,55]. As personalized, non-standardized medical devices, these appliances currently lack established design or manufacturing quality controls beyond basic material approval. Ensuring safety is therefore paramount: before adopting new workflows, cleft centres must prioritize patient protection and address potential risks.

In addition to the safety wire, the authors recommend a static, non-adjustable design, avoiding dynamic systems such as those proposed by other centres [48,49,51,52,53], where the angle or position of the extension can be modified using screws, wires, thermoplastic materials, or orthodontic components (Table 2). When applying dynamic setups in the prototyping stage, neither the accuracy nor the safety of transferring prototype adjustments to the final extension has been addressed [54]. Although these dynamic designs aim to simplify fitting and allow easier adjustments during treatment, they reduce control over the approved configuration once the patient is outside clinical supervision. It remains uncertain what may happen if caregivers unintentionally alter the setup—for example, unintentional dropping of the appliance while cleaning, by stepping on the appliance or using it incorrectly. Any post-discharge changes must be either impossible or easily detectable. Such modifications might be harmless, but may also lead to oral lesions on the tongue base or posterior pharyngeal wall, recurrence of UAO, or other complications—presenting with symptoms similar to those seen in poorly fitted TPPs, such as new-onset swallowing difficulties, fever, or restlessness [7]. Most reported approaches rely on dynamic setups—sometimes even single-rod appliances or screws—for both prototyping and the final TPP, often without a safety wire. Whether such designs are truly safe remains unproven. By contrast, the static, non-adjustable design with an integrated reinforced safety wire, as presented here, ensures stability and safety during endoscopy and after discharge. Any extraoral alteration would be immediately evident, as the rigid, non-flexible material would fracture if tampered with. For these reasons and given the lack of rigorous safety evaluation in the literature, we strongly advocate for a fixed design that eliminates the possibility of bending, positional changes, or unintentional caregiver modifications, thereby offering superior stability and patient safety.

**Figure 25:**
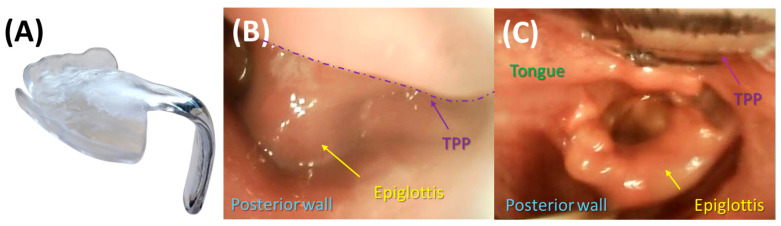
Use of a transparent TPP prototype for endoscopic fitting. (**A**) Fully post-processed transparent prototype manufactured via AM. (**B**) Endoscopic view in which the distal end of the extension is not clearly discernible due to low contrast. (**C**) Endoscopic view with the distal end marked using a black marker to enhance visibility (note a malformed epiglottis and asymmetrical pharynx in this syndromic patient).

Moreover, transparent materials have been proposed for prototyping and final TPP (Figure 25A) [22,48,50,53,55]. Transparent materials can significantly hinder endoscopic assessment, particularly during the initial learning phase, making evaluation and modifications more challenging. Additionally, in patients with high saliva or mucus production, where visibility may be compromised, a high-contrast appliance is essential for ensuring optimal contrast against the mucosa (Figure 25B). The main reason is that most MDR Class IIa splint materials required for the final TPP—and available on the market for both AM and subtractive manufacturing (SM)—are primarily offered in transparent form, as this appeals to a broader market. Opaque or coloured Class IIa materials are either unavailable or lack commercial viability. Although contrast-enhancing solutions, such as marking the extension tip with a black marker, have been proposed (Figure 25C), the resulting contrast and usability remain inferior to that achieved with high-contrast opaque colours (Figure 14). However, MDR Class I materials—suitable for endoscopic use due to limited mucosal contact (<24 h), but unsuitable for final appliances—offer a wider range of options. Prior to adopting the endoscopic material proposed in this study, in vitro tests on mechanical strength and safety were conducted, confirming its suitability for prototyping and endoscopic evaluation [24]. Furthermore, the recommended prototyping material has been shown to be sufficiently safe [24], eliminating the need for wires and allowing for streamlined bedside modifications, such as shortening. Equally important, the final TPP, like the one recommended here, also comprises a blue extension.

## 8. Special Velopharyngeal Extension Configurations for Non-Standard RS Cases

### 8.1. No Hard and Soft Cleft Palate

Most RS patients have both soft and hard palate involvement [12,58]. However, some patients may present with only uvular or soft palate clefts, and in rare cases, no cleft is observed. Despite this variation, TPP treatment can still be effective, even in the absence of a cleft. Nevertheless, this presents additional challenges in configuring the extension and performing the necessary endoscopic evaluation.

Regarding the prototyping and final manufacturing of the TPP, the same process described earlier is followed. The main difference lies in the standard velopharyngeal extension used to create the three prototypes, which has a different shape. Specifically, the velopharyngeal extension features a flatter cleft extension part, as shown in Figure 7D–G. This design ensures additional spacing between the palatal cavity and the posterior maxillary region, which is crucial for proper function. Patients without a cleft typically have less space compared to standard RS cases with both soft and hard palate involvement. Without this additional spacing, the TPP may contribute to increased UAO or interfere with effective feeding. Furthermore, there is a substantial risk of pressure sores in the uvular region when no cleft is present.

In terms of nasopharyngeal endoscopy, the reduced space caused by the absence or minimal size of the cleft limits the space for positioning the endoscope tip. It becomes more difficult to achieve the optimal angle for visualizing the velopharyngeal extension’s position within the pharyngeal cavity and its relation to the tongue and epiglottis. As a result, evaluating the prototypes is more challenging if the cleft is small or absent. This challenge is further compounded if the patient has a hypotonic pharyngeal musculature (Sher type II or III) or other complications, which are typically seen in syndromic RS cases.

### 8.2. Hypotonic Oropharyngeal Musculature and/or Challenging Anatomical Structures

**Figure 26:**
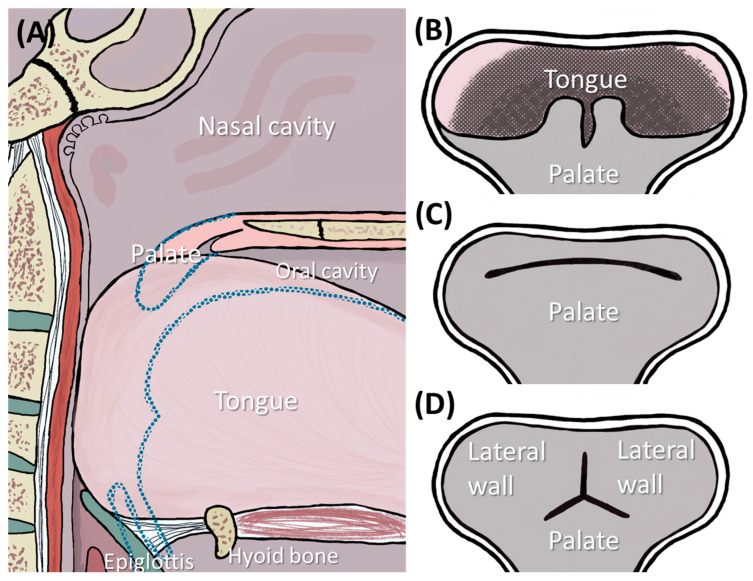
Schematic illustrations based on the Sher classification [36]: (**A**) sagittal view showing Sher Type I: the tongue lies against the posterior pharyngeal wall, displacing the soft palate and epiglottis. The blue line indicates the normal positions of these structures, allowing an open airway. (**B**) Endoscopic view from the nasal cavity (Type I): the cleft palate (grey) and tongue (brown) are seen; the tongue adheres to the posterior pharyngeal wall, narrowing the airway. (**C**) Endoscopic view of Type II: the tongue displaces the soft palate against the posterior pharyngeal wall, causing oropharyngeal narrowing involving the tongue, the pharyngeal walls, and the velum. (**D**) Endoscopic view of Type III: the lateral pharyngeal walls collapse medially, meeting the soft palate and constricting the oropharynx.

Upper airway collapse may result not only from glossoptosis but also from hypotonic oropharyngeal muscles and abnormal anatomy, especially in RS patients with syndromes or craniofacial disorders such as craniosynostosis. Sher et al. [36] classified obstruction severity during nasopharyngoscopy into four types (I–IV) (Figure 26). Type I, the mildest and most common within this classification, involves the tongue falling against the posterior oropharyngeal wall (Figure 26A,B). Type II features the tongue pressing the posterior pharyngeal wall without touching the soft palate (Figure 26C). Both types respond well to standard or simple TPP treatment.

**Figure 27:**
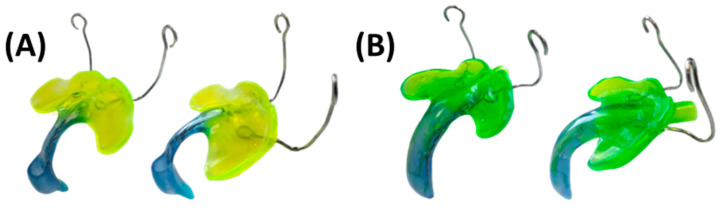
Isometric and lateral views of various special configurations of the velopharyngeal extension in TPP designs. (**A**) Ring TPP: featuring a ring-shaped structure in the lower third of the extension. (**B**) Flute TPP: incorporating a tubular extension that begins extraorally and runs along the entire length of the extension.

However, if the airway collapse originates from the lateral oropharyngeal walls (Sher Type III or IV), the TPP extension must deviate from the standard design, requiring a more complex and prolonged fitting process. In Sher Type III, the lateral pharyngeal walls collapse (Figure 26D). To counter this, a ring can be added to the plate’s pharyngeal extension (Figure 27A) to help mantain the airway open [59]. This solution requires a palatal cleft for safe insertion; without it, there is a higher risk of injuring the uvula and velum, particularly during plate removal. Careful quality control is essential: the ring wall must not be too thin to prevent cracking, and it must be thoroughly polished to avoid damaging the delicate pharyngeal mucosa.

Type IV involves a circular, sphincter-like collapse of all upper airway structures, often due to tight anatomy such as a hypoplastic midface seen in craniosynostosis. This severe obstruction is addressed with a flute TPP—a rigid, tubular extension bridging the entire pharynx and acting as a nasopharyngeal airway (Figure 27B) [59]. Despite its complexity, patients over one year of age often tolerate the flute TPP well, using it as a night-time alternative to high-flow therapy. A cleft palate is not required for this device, which is advantageous.

**Figure 28:**
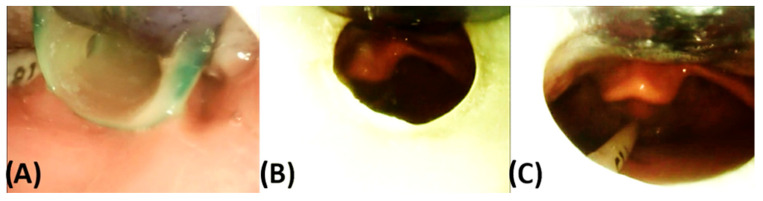
Nasopharyngeal endoscopy of a patient with a ring TPP: (**A**) Cranial entrance of the ring, showing a suction catheter on the left and a nasogastric tube (white). (**B**) Internal view of the ring. (**C**) View through the end of the ring showing the epiglottis and allowing assessment of the extension´s length.

For both ring and flute TPPs, side openings are essential to allow secretion drainage (Figure 28). The diameter must conform to the pharyngeal shape and allow passage of a suction tube to clear viscous secretions, ensuring proper ventilation and preventing airway blockage. It should also be large enough for a nasopharyngeal endoscope. Awake nasopharyngeal endoscopy is critical for fitting and adjusting the extension to the collapsing structures and monitoring the airway’s response (Figure 28). Notably, these TPP modifications have proven successful in patients with severe midface hypoplasia, such as Apert or Crouzon syndrome. Clinical experience indicates that primarily night-time application is well-accepted, particularly by older patients and contributes to therapeutic success.

Despite this, the authors emphasize that such specialized appliances have been used in only a very small proportion of RS patients at our centre and are reserved exclusively for cases in which the standard TPP proved insufficient. Their use requires a highly experienced multidisciplinary team and appropriate infrastructure, given the complexity of the fitting process and the necessity of endoscopic assessments (Figure 28). Additionally, the multiple required modifications and the associated workload in the dental laboratory are time-consuming and cumbersome. Although previous studies have reported successful outcomes and confirmed their safety [12,59], we have encountered emergencies—such as UAO due to mucus-induced obstruction of the ring or flute extension—even under close clinical supervision. As a result, our centre has discontinued their use over the last four years, mainly for safety concerns, and we are increasingly using high-flow nasal cannula therapy instead in these syndromic patients. These devices demand substantial clinical expertise and caregiver commitment and should be considered only when standard TPP treatment fails—keeping in mind that safety cannot be guaranteed. Despite isolated positive outcomes, we strongly advise against their use, particularly in less experienced centres.

For such patient cases, our centre typically uses a standard TPP, often combined either intermittently (e.g., at night) or continuously with high-flow therapy, depending on sleep laboratory findings. Additionally, RS cases without a cleft are treated non-invasively with high-flow therapy alone, as our centre currently does not pursue surgical alternatives such as MDO.

## 9. Subsequent TPPs

Due to rapid maxillary growth, the first TPP often becomes insufficient after 2–3 months, particularly in newborns initially weighing < 3 kg [7]. Clinical signs indicating the need for a second TPP include loosening or detachment of the appliance, an infant attempting to remove it, or the appearance of increased dents in the mucobuccal fold (as explained above), suggesting the plate rim has become too small (Figure 21A,B) [14]. While usually non-painful, such impressions may signal inadequate fit, though they rarely cause mucosal inflammation.

For a subsequent plate, the patient and their caregivers are readmitted to hospital, using family rooms located within the department of neonatology. The previous plate is kept outside the mouth for approximately 48 h, to provide some relief to the mucosa and to allow the indentations from the previous plate to recede. Patients both at home and within this time period without TPP are monitored by a mobile pulse oximeter. Once the mucosa has no indentations, a maxillary scan is performed by the orthodontists.

**Figure 29:**
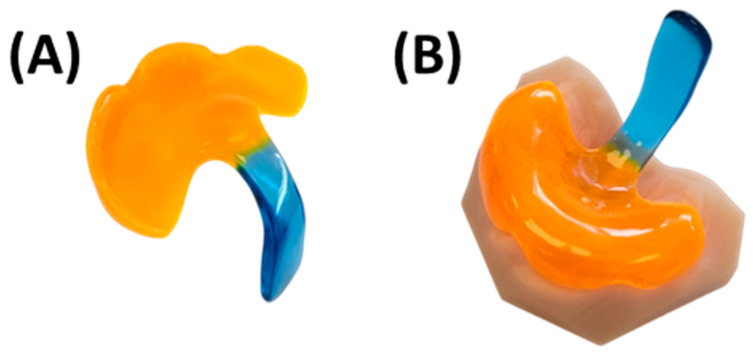
Conventionally manufactured prototype for a subsequent TPP. (**A**) Top view of the prototype. (**B**) Prototype positioned on the corresponding additively manufactured maxillary model.

In contrast to the initial TPP, the fabrication of subsequent plates benefits from prior knowledge regarding the optimal configuration of the velopharyngeal extension. As this information is already available, it is sufficient to additively manufacture a new maxillary model based on the latest intraoral scan. Producing multiple prototypes at this stage is redundant and time-intensive, given that a functional and clinically successful TPP already exists for the patient. Accordingly, a single, manually fabricated TPP is created by the dental technician using the newly printed model and the previously used TPP. The same configuration of the velopharyngeal extension is transferred, creating a new base plate. The dental technician measures the growth in the sagittal dimension on the model, sets the first extension in relation and shifts it posteriorly. This usually is about 1–1.5 mm posteriorly. Further, the extension is lengthened by approximately 4 mm. Once the appropriate position of the extension is set, the TPP prototype is manufactured as previously described directly from PMMA Orthocryl (Dentaurum). For this manual TPP prototype, the extraoral fixation bows and wires are initially not attached (Figure 29).

Next, an endoscopy is typically performed the morning after imaging in the department of neonatology (Figure 2 and Figure 3). On the day of the procedure, the patient should remain unfed for approximately two hours. During endoscopy, the extension’s position is assessed, and potential adjustments are discussed by the interdisciplinary team. Parents are encouraged to attend, actively engage in the discussion, and contribute their practical experience with the original TPP used at home. Having spent time with their baby, parents often provide valuable insights into the effects of plate therapy. Their observations—such as changes in drinking behaviour, breathing patterns, and how these evolved over time with the progressively smaller plate—can be particularly helpful.

Once endoscopic feedback is obtained, the prototype is returned to the dental laboratory. In most cases, the second fitting is smooth and requires no major modifications. If no significant adjustments are needed, the dental technician integrates the safety wire and extraoral fixation bows, performing any minor refinements as necessary. During this appointment, the fixation bows are individually adapted to the patient’s anatomy. The appliance is then inserted using adhesive cream and externally secured with adhesive tapes.

Following insertion, the patient’s tolerance of the new appliance is observed clinically, following the same protocol employed in the first plate.

Generally, the adjustment of the subsequent TPP takes 5–7 days from admission. Because patients and parents are already familiar with the TPP, the adaptation of the patient-individualized extension is easier to confirm. The patient can be discharged if the following criteria are met: (1) Polysomnography shows an OAI < 3/h, (2) no respiratory noises, (3) sucking and swallowing are possible, and (4) the mucosa shows no irritation. The next appointment is scheduled for 6 weeks after discharge at the Department of Orthodontics to monitor the second TPP.

As full disclosure, it should be noted that in recent years our centre has not routinely provided a second plate for all patients. This is because the majority have shown such favourable progress after the initial plate that a second plate is often not indicated. When the mandible is well-positioned, the tongue lies anteriorly, and sleep laboratory results are favourable, TPP treatment is considered successful and subsequently discontinued. The long-term impact of this shortening of TPP therapy remains to be determined, as this change was only introduced in recent years with the aim of reducing the treatment burden for both parents and patients. In cases where a second TPP is not required based on these objective parameters, centres may also benefit from a reduced workload, thereby facilitating the broader implementation of TPP therapy in other institutions.

After approximately 3–4 months of using the second TPP, a follow-up appointment is scheduled to conduct a polysomnography without the appliance, in order to assess whether sleep parameters remain within the normal range in its absence. This scheduling is initiated once caregivers report that the appliance has become too small. Then, a sleep study appointment is scheduled. To eliminate potential carry-over effects, parents are instructed to discontinue TPP use for 7–10 days prior to the study. If the sleep study confirms an OAI < 3/h, TPP therapy is discontinued. In rare cases where the OAI remains > 3/h, a third TPP is fabricated, and the described workflow is repeated.

## 10. Other TPP Treatment Success Factors

In addition to a well-fitted, individually tailored TPP appliance, several other factors play a decisive role in treatment success. Foremost among these is strong parental involvement. Caregivers must actively participate in all aspects of therapy, including appliance placement, maintenance, monitoring for issues, proper taping, and feeding training. Specialized nurses are also essential, as they play a key role in supporting and encouraging parents to continue the therapy without becoming discouraged by potential setbacks or the child’s distress.

Equally essential is the implementation of orofacial regulation therapy—not only to improve sucking and feeding behaviour, but also to strengthen the orofacial musculature and stimulate mandibular growth.

Adherence to therapy is another key determinant. The TPP should be worn continuously, except during cleaning or if otherwise clinically indicated. In rare cases, the appliance is worn only during sleep, with a stimulation plate used while awake.

Regular and well-timed follow-up appointments with the multidisciplinary team are also critical. These visits allow for monitoring of progress, early detection of complications, and continued support for caregivers through reinforcement of training and clarification of any concerns.

Finally, prenatal diagnosis and prompt referral to the cleft centre increase the likelihood of timely therapy initiation, improving the child’s chances of successful adaptation.

## 11. End of Treatment and Next Stages

Typically, two TPP appliances (rarely three) are needed to continue therapy until the infant is approximately 6–9 months old [7]. Treatment is considered complete once a positive sleep study confirms an OAI < 3. In rare cases, therapy may be discontinued prematurely due to poor acceptance by the patient or caregivers, complex anatomical conditions often seen in syndromic RS, or unrelated medical issues (e.g., surgeries). In such instances, securing the upper airway remains the priority, and alternative treatment options should be explored, such as the use of a feeding plate or cleft-covering palatal plate in combination with high-flow therapy.

If TPP therapy is not initiated immediately after birth, teething may begin during treatment. The eruption of anterior deciduous teeth usually does not interfere, as the palatal baseplate can be modified accordingly. However, the eruption of multiple posterior teeth—such as molars—prevents secure fitting of the appliance due to insufficient palatal support, necessitating early discontinuation. This is generally not problematic, as teething often begins after the optimal treatment window and typically starts in the lower jaw, by which time airway-related issues have usually resolved.

**Figure 30:**
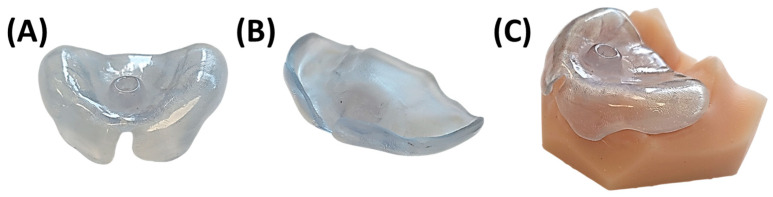
Stimulation plate post-TPP therapy: (**A**) Top view of the finished plate. (**B**) Unpolished palatal side for secure fit. (**C**) Appliance on its corresponding maxillary model.

Once TPP therapy is discontinued and a sleep study confirms the absence of UAO, progression to stimulation therapy based on the Castillo-Morales orofacial therapy is generally recommended. While not all children require stimulation therapy, those who do are treated using a previously established fully digital workflow to design a patient-specific palatal plate (Figure 30) [21,32]. This appliance resembles presurgical plates used in cleft lip and palate patients but includes an anterior or medial stimulation element. The element promotes active tongue movement, supports anterior tongue positioning, and encourages mandibular advancement [27,28,29]. Additionally, the plate helps close the cleft palate, separating the oral and nasal cavities to facilitate feeding. It is typically worn for 3–4 h per day. In cases where feeding is impaired due to the cleft, a version without the stimulation element may be used [32].

About 5–6 months following successful TPP therapy, surgical closure of the cleft palate is performed. A positive sleep study showing an OAI < 3/h is required beforehand, as the surgery may narrow the airways again. Due to the cleft, there is an increased risk of recurrent otitis media with effusion, which can impair hearing and subsequently affect language development [60,61]. Therefore, routine preoperative otological and audiological assessments are carried out in collaboration with otorhinolaryngology specialists. If effusion is detected, ventilation tube placement or paracentesis can be performed concurrently with cleft palate surgery. This interdisciplinary approach helps minimize the number of anesthetic procedures required in these young patients.

A cleft palate can lead to velopharyngeal insufficiency, defined by inadequate closure between the nasopharynx and the oropharynx. This may result in nasal regurgitation and imprecise or hypernasal speech. The TPP approach therefore includes regular interdisciplinary follow-up care with specialists in speech therapy, oral and maxillofacial surgery, otorhinolaryngology, and orthodontics. These evaluations enable timely adjustments to the treatment plan in close coordination with patients and their caregivers.

Continuous monitoring of sleep-disordered breathing is recommended throughout growth. Our protocol follows this schedule: every 3 months during the first 3 months after surgery, then annually until age six, and thereafter every two years. In rare cases, obstructive sleep apnoea may persist in RS patients despite TPP therapy, usually affecting those with complex RS cases involving syndromes. In such situations, nocturnal respiratory support is required and should be managed through long-term follow-up [62].

## 12. Orthodontic Treatment Considerations Following TPP Therapy

In patients without craniofacial disorders, orthodontic treatment typically begins in the primary dentition during preschool age, depending on patient compliance. In contrast, individuals with RS require earlier orthodontic management due to their characteristic craniofacial growth patterns and dental anomalies [63]. RS is associated with skeletal growth disturbances, including bimaxillary retrognathia, an increased mandibular plane angle, and a shortened ramus, resulting in skeletal Class II malocclusion. Intraoral findings commonly include severe crowding, bifrontal lingual inclination of the anterior teeth, and bimaxillary arch constriction [64,65,66,67,68].

Despite successful early TPP therapy, patients with RS require extensive long-term orthodontic treatment [15]. Treatment is delivered in phased stages aligned with dentition development (Table 3) and serves as a central hub for interdisciplinary coordination with oral and maxillofacial surgery, speech therapy, and other specialties. The primary objectives are to correct malocclusion and skeletal Class II discrepancies while restoring optimal stomatognathic function. Regular interdisciplinary consultations during key growth phases are essential to optimize speech, function, and facial esthetics. Following comprehensive orthodontic rehabilitation, lifelong retention and ongoing interdisciplinary follow-up are crucial to maintain results.

## 13. Research Supporting the TPP Approach from Our Centre

Since the development of the TPP approach in Tübingen, multidisciplinary studies have been published (Table 4 and Table 5). In this way, the TPP is a validated, minimally invasive treatment for infants with RS, effectively relieving UAO, promoting mandibular growth, and improving feeding outcomes [5,9,11,57,69]. Longitudinal studies show normalized jaw and airway parameters, catch-up growth, reduced need for tube feeding, and favourable neurocognitive and speech development following early TPP use [10,15,70,71]. Compared to surgical alternatives, TPP reduces perioperative risk and is particularly advantageous for early airway and feeding management [72,73]. Advances in IOS and CAD/CAM technology have improved the safety, precision, and efficiency of TPP production [18,21,22,23,24,25]. Overall, TPP supports a conservative, multidisciplinary approach and remains an effective first-line treatment in RS care [1,7,12,74].

While numerous publications from our centre confirm the effectiveness of the TPP (Table 4 and Table 5), this section focuses on the most debated aspects of TPP treatment, e.g., its effectiveness in comparison to alternatives such as mandibular distraction osteogenesis (MDO). A recent study by Resnick et al. demonstrated that both MDO and TPP effectively alleviate UAO and improve feeding and growth in infants with RS [73]. While MDO generally achieved immediate resolution of UAO, TPP was associated with more favourable early outcomes in feeding and weight gain. The superiority of MDO in airway management was most evident in infants with severe pre-intervention UAO. Based on these findings, and in line with the principle of prioritizing non-surgical approaches when outcomes are comparable, the study suggested TPP as a suitable option for infants with moderate UAO, while MDO may remain the first-line treatment for severe cases [73]. In our experience, however, the effect of TPP treatment on sleep-related UAO is most pronounced—and most rapid—in infants with the most severe pre-treatment sleep study results [57]. Additionally, the TPP has been recommended by the European Reference Network (ERN) for Craniofacial Anomalies guidelines as the primary non-surgical approach. It is advised to be used before considering surgical interventions, with surgery only being pursued if the less invasive method proves unsuccessful [75].

One of the main critiques of the TPP approach concerns its growth-promoting potential compared to MDO, with much of the research community favouring MDO in this regard. However, short-term data suggest otherwise: a previous study of 31 infants treated with the TPP showed a significant improvement in Jaw Index within the first three months [11]. Another study comparing 19 RS patients to 32 healthy controls demonstrated significant catch-up growth in TPP-treated infants, evidenced by reductions in both Jaw Index and ANB’ angle [69]. Although initial differences were evident, both measures showed continuous improvement over time, pointing to a gradual normalization of the facial profile within the first year [69]. These results imply that TPP may contribute to mandibular growth within a non-surgical treatment framework.

Meanwhile, considering long-term functional impact of the TPP, a study evaluating the long-term effects of TPP treatment in school-aged children with RS found that functional and dentoalveolar outcomes during school age were comparable to, or even better than, those of healthy peers [9]. Skeletal measurements remained within normal limits, and lateral cephalometric analysis showed that the posterior airway space (PAS) was equal to or greater than that of non-RS controls, indicating no UAO [9]. The lasting postnatal effects of TPP treatment are attributed to its functional design, which applies gentle mechanical forces that promote anterior tongue positioning and mandibular advancement. These physiologic movements stimulate condylar growth, contribute to airway expansion, and support the functional reorganization of the stomatognathic system [9]. This aligns with Roux’s “form follows function” principle, highlighting that morphological development is driven by functional adaptation [13]. While bone development was favourable, soft tissue adaptation was less complete, leading to a slightly more convex facial profile and reduced lower face height [9].

However, it is important to emphasize that the success of this therapy does not rely solely on the use of the personalized TPP appliance. The Tübingen treatment concept is built on two pillars: the TPP and the non-appliance-based myofunctional therapy. This involves targeted exercises to normalize orofacial functions and improve muscular tone, implemented at Tübingen through orofacial regulation therapy based on the Castillo-Morales concept [31,76] and carried out by both, speech therapists and neonatal nurses. Myofunctional therapy is intended to continue beyond the initial treatment phase, throughout the child’s growth, and in parallel with orthodontic follow-up. The therapeutic plan should be regularly adjusted based on the child’s progress and compliance, as assessed by a speech therapist.

As mentioned before, despite clear improvements in functional parameters such as dental arch width and PAS during infancy and school age, esthetic limitations remain [9]. Specifically, full mandibular projection is often not achieved [9]. Additionally, certain RS-related issues such as specialized craniofacial anatomy [77], dental anomalies [15], and an increased risk of sleep-disordered breathing persist despite early intervention [62].

With regard to the growth pattern of patients with craniofacial disorders during this age period, the results demonstrated comparable growth characteristics and facial morphology between patients with RS and those with an isolated cleft palate [78,79]. However, mandibular length remained significantly shorter in the RS group compared to individuals with an isolated cleft palate. Hotz and Gnoinski confirmed this correlation between growth pattern and the underlying primary anomaly—such as a cleft palate—which manifests not only during infancy, but also beyond [80]. Nevertheless, within the first two years of life, the mandibular dimensions of children with RS do not reach those of unaffected children [81]. Concerning the possibility of mandibular catch-up growth during preschool and school age following conservative treatment, the characteristic convex and retrognathic facial profile of RS patients—reflecting a skeletal and dentoalveolar Class II malocclusion—remains apparent and typically persists beyond the age of five and into adulthood [64,65,66,82].

Thus, the literature shows that despite successful TPP therapy or other treatment options, primary symptoms of RS persist during growth and are clearly reflected in the growth pattern. Differences in global treatment concepts, the low prevalence of RS, small patient numbers at individual centres, and heterogeneous study designs make meaningful comparisons difficult. In particular, there is a lack of long-term data on the impact of surgical treatment on growth during childhood and beyond.

In summary, the primary goal of any treatment for RS is to address the immediate challenges of feeding and breathing. While MDO may be appropriate in syndromic cases with severe UAO, the TPP offers a less invasive alternative with proven benefits, not only in aiding UAO; but also in improving feeding, supporting weight gain, promoting mandibular growth, and reducing surgery-related complications. These outcomes provide strong justification for considering TPP as the first-line treatment in most RS cases.

**Table 4:** Part 1—multidisciplinary evidence supporting the TPP approach (2025–2021).

Authors	Shortened Title	Key Findings	Main Topics/Disciplines
Weismann et al. (*Under review*) [40]	Orthodontic Appliance-Related MUs in Infants with Craniofacial Disorder (CD)	In patients with palatal plates and CD were evaluated, where 88% of RS patients developed MUs. Plate type significantly influenced rates; however, sex, cleft location, syndrome, and morphology did not.	Orthodontics, Craniofacial Anomalies, Mucosal Pathology
Wiechers et al. (2025) [83]	Positioning and OSA in RS	Prone position improves but does not eliminate OSA and may worsen symptoms in some cases.	Sleep Medicine, Pediatric OSA, Positioning Therapy
Wiechers et al. (2024) [69]	Facial Profile Changes in RS with TPP and Controls	TPP supports mandibular catch-up growth; ANB’ angle and Jaw Index improved over time.	Orthodontics, Craniofacial Growth and Development
Wiechers (2023) [62]	Sleep and neurocognitive outcomes in primary school children with RS	Children with RS had higher risk of OSA and behavioural problems, while neurocognitive outcomes were normal. Regular OSA screening throughout childhood is recommended.	Sleep Medicine, Pediatric OSA
Effert et al. (2023) [9]	Prospective Orthodontic Evaluation Post-TPP	School-aged RS children post-TPP showed normal skeletal/dentoalveolar parameters, PAS, with stimulated mandibular/TMJ (temporomandibular joint) growth but a more convex soft tissue profile.	Orthodontics, Craniofacial Growth, Airway Assessment
Knechtel et al. (2023) [14]	Caring for Infants with TPP	Practical tips from 20 years’ experience on TPP feeding, cleaning, and placement.	Nursing Practice, Feeding Management, Medical Device Care
Effert et al. (2023) [15]	Orthodontic Needs after TPP	RS patients show higher orthodontic needs regardless of cleft status.	Orthodontics, Craniofacial Anomalies
Oechsle et al. (2022) [74]	Multicentre Registry for RS Treatment	Prospective multinational registry collects standardized data on treatments, complications, and outcomes to improve personalized care and long-term understanding.	Clinical Research, Multicentre Registry, Evidence-Based Medicine
Aretxabaleta et al. (2022) [8]	CAD Measurement semi-automation for TPPs	CAD-based semi-automatic method enables fast, accurate, reproducible TPP measurements.	Digital Dentistry, CAD/CAM Technology, Orthodontics
Naros et al. (2022) [70]	Neurocognitive Development in Isolated RS Treated with TPP	Children with isolated RS treated early with TPP showed normal cognitive development by age 5–6, comparable to cleft-only peers.	Pediatric Neurodevelopment, Craniofacial Anomalies, Early Intervention
Naros et al. (2022) [71]	Speech Development in Cleft Palate with/without RS	Good speech outcomes in RS and cleft-palate-only children; isolated RS, surgery timing, and cleft severity did not worsen results after TPP treatment.	Speech–Language Pathology, Craniofacial Speech Outcomes
Resnick et al. (2025) [73]	Comparison of TPP and MDO	Both MDO and TPP improve airway, feeding, and growth; MDO superior for severe UAO, whereas TPP shows better early feeding and weight gain.	Comparative Treatment Outcomes, Airway Management, Feeding, Craniofacial Surgery
Wiechers et al. (2021) [7]	Evidence and Practice with TPP	Review highlighting clinical effectiveness and practical aspects of TPP treatment.	Clinical Review, Orthodontics, Craniofacial Treatment
Wiechers et al. (2021) [10]	Growth Outcomes after TPP in RS	Catch-up growth after initial weight loss; tube feeding decreased post-TPP.	Feeding Management, Growth Monitoring, Craniofacial Treatment
Naros et al. (2021) [72]	Perioperative Complications in RS Cleft Palate Repair Post-TPP	Early TPP treatment corrects UAO and reduces perioperative complications in RS cleft palate repair.	Surgery, Perioperative Care, Airway Management
Aretxabaleta et al. (2021) [25]	Accuracy of Additive vs. Subtractive Palatal Plates	Subtractive methods showed higher accuracy than additive ones; DLP at 100 µm balances accuracy and efficiency for TPP production.	Manufacturing Technology, Digital Dentistry, Material Science

**Table 5:** Part 2—multidisciplinary evidence supporting the TPP approach (2021–2007).

Authors	Shortened Title	Key Findings	Main Topics/Disciplines
Aretxabaleta et al. (2021) [24]	Fracture Load of Digital TPP Appliances	CAD/CAM improves TPP production; Freeprint tray recommended for prototyping, Smile PEEK for the final TPP for maximum safety.	Materials Science, Device Safety, Digital Manufacturing
Aretxabaleta et al. (2021) [23]	CAD/CAM Materials for TPP: Flexural Strength Study	SLA and SM methods had the highest flexural strength under standardized testing.	Material Testing, Digital Dentistry, Biomechanics
Wiechers et al. (2021) [84]	Treatment of Infants with Craniofacial Malformations	Infants with CD/RS at high risk for respiratory, feeding, and developmental issues requiring early multidisciplinary care and prenatal planning.	Multidisciplinary Care, Neonatology, Craniofacial Medicine
Weise et al. (2021) [18]	IOS of Neonates and Infants with Craniofacial Disorders	IOS is a fast, safe, feasible procedure for neonates and infants with craniofacial malformations like RS.	Digital Dentistry, Clinical Workflow, Pediatric Care
Xepapadeas et al. (2020) [22]	Digital Workflow for RS: Technical Note Part II	Digital workflow enabled successful TPP prototype production from intraoral scans.	Clinical Workflow, Digital Dentistry, Orthodontics
Müller-Hagedorn et al. (2020) [85]	TPP Prototype Method	Thermoplastic adjustable spur improves airway and stimulates mandibular growth.	Orthodontics, Device Design
Xepapadeas et al. (2020) [21]	Digital Workflow for Trisomy 21 Plates: Technical Note Part I	Workflow for base palatal plate production.	Clinical Workflow, Digital Dentistry
Wiechers et al. (2019) [11]	Mandibular Growth in Infants Treated with TPP	Jaw Index and apnoea scores improved; tube feeding dropped from 84% to 8%; no craniofacial surgery needed; mandibular growth promoted.	Orthodontics, Feeding Management, Craniofacial Growth
Poets et al. (2019) [12]	Summary of Current Evidence on TPP	TPP effective in relieving UAO, stimulating growth, and improving cleft outcomes; alternative to surgery.	Review, Airway Management, Craniofacial Treatment
Müller-Hagedorn et al. (2017) [59]	TPP for Syndromic RS	Modified TPP designs including ring and tube solutions proposed.	Orthodontics, Syndromic Craniofacial Disorders, Device Innovation
Poets et al. (2017) [5]	Multicentre Study on PEBP/TPP	PEBP treatment significantly reduced apnoea index and oxygen desaturation; tube feeding dropped from 74% to 14% with stable weight.	Multicentre Clinical Study, Airway Management, Feeding
Buchenau et al. (2017) [4]	Functional Treatment Using PEBP/TPP	PEBP reduced apnoea index, improved weight gain, and lowered tube feeding in isolated RS without surgery or tracheostomy.	Airway Management, Feeding, Non-invasive Treatment
Maas et al. (2014) [86]	Prospective Study on Initial Treatment and Early Weight Gain in Germany	Prone positioning (61%) and functional therapy (57%) common; PEBP used in 23%; surgery rare (6%), favouring non-invasive treatments.	Epidemiology, Treatment Patterns, Feeding Management
Vatlach et al. (2014) [1]	Birth Prevalence and Treatments in Germany	RS prevalence 12.4/100,000; majority treated conservatively; few required surgery.	Epidemiology, Treatment Trends
Poets et al. (2011) [87]	Treatment of UAO and Feeding in RS-Like Phenotype	Overview of treatment strategies for RS-like conditions.	Treatment Strategies, Airway and Feeding Management
Bacher et al. (2010) [6]	Treatment of Infants with RS	Overview of conservative vs. surgical options; PEBP introduced as an effective non-invasive alternative.	Airway Management, Feeding, Treatment Options
Buchenau et al. (2007) [57]	Randomized Trial of New Orthodontic Appliance for RS	Appliance with velar extension reduced apnoea index by 71% without adverse effects.	Sleep Medicine, Orthodontics, Clinical Trial
Bodman et al. (2003) [16]	The Tübingen palatal plate, an innovative therapy concept for RS (*in German*)	Appliance with extension effectively treats UAO, reducing hypoxemia and supporting mandibular growth	Sleep Medicine, Neonatology, Orthodontics, Clinical Trial

While less invasive yet effective techniques, like the one proposed here, are gaining international acceptance, the management of RS still requires a multidisciplinary team. Given the rarity of RS, centralizing treatment within each country is advisable. Centralized centres provide access to specialized expertise, comprehensive care, and efficient resource use. They enhance research through clinical trials and focused studies, reduce redundant infrastructure, and ensure consistent, high-quality care via standardized protocols. Such centres also support patient networks, streamline regulatory processes, and improve long-term outcomes through continuous care and international collaboration. Ultimately, centralization fosters better health outcomes and advances in treatment and research for rare diseases. Usually, comparisons across studies are limited by treatment heterogeneity, small cohorts, and inconsistent designs, with long-term surgical outcome data especially scarce. These challenges call for specialized, interdisciplinary care in centres with standardized protocols. The ERN Cranio is addressing this need through multicentre collaboration, larger cohorts, and patient involvement to unify and improve RS care across Europe [75]. This has already been undertaken in our centre through participation in a multicentre registry on RS treatments [74].

## 14. Conclusions

The TPP offers a validated, minimally invasive option for managing UAO and feeding difficulties in infants with RS, while also supporting physiological mandibular growth and neuromuscular development during a critical developmental window. This technical note outlines a semi-digital, interdisciplinary protocol developed at Tübingen University Hospital, detailing each step from IOS to final appliance delivery and follow-up.

By integrating CAD/CAM technologies with conventional dental fabrication, this workflow ensures precision and adaptability while maintaining clinical flexibility for individualized care. The use of standardized digital velopharyngeal extensions, validated endoscopic fitting procedures, and stringent quality control measures ensure safety and reproducibility across cases. Its semi-digital nature improves efficiency and reduces errors in manufacturing, while remaining feasible for routine clinical use. Importantly, safety considerations—such as the mandatory use of a safety wire and static appliance design—are emphasized, particularly in light of the lack of mechanical testing or safety standards in many reports on alternative approaches.

Beyond direct patient care, the digital components of this workflow—such as IOS and CAD data—offer significant advantages for training, inter-centre collaboration, and data sharing. These features support the development of multicentre, prospective studies, enabling the generation of robust, evidence-based data and the establishment of treatment standards for RS. The digitalization of key steps also allows for remote consultations, improved interdisciplinary coordination, and broader implementation in other qualified cleft centres worldwide. Given the rarity of RS, the centralization of care within specialized centres is strongly advocated. Such structures allow for consistent high-quality care, concentration of clinical expertise, and optimal use of healthcare resources. They also provide ideal conditions for research, education, and long-term outcome tracking.

In conclusion, this study serves not only as a step-by-step technical guideline, but also as a framework for the safe and efficient clinical implementation of TPP therapy in RS. It underscores the importance of standardization, safety, and interdisciplinary collaboration in the treatment of rare craniofacial anomalies and sets the stage for future advancements in both clinical practice and scientific research.

## Figures and Tables

**Table 1:** Checklist summarizing key fabrication steps and follow-up information.

Main Step	Substeps	Checklist Item
**Scanning**		- Capture at least two-thirds of tuber region. - Maximize vestibular region; scan later if access is limited. - Avoid artefacts; use software correction or rescan. - Evaluation of physiological intraoral mucosal conditions. - In case of MUs, dents, or injuries, wait for complete healing before scanning.
**TPP** **prototyping**	Design and manufacturing	- Share key clinical info, including patient age, with the technical team. - Note intraoral features affecting IOS or TPP design. - Start with a simple design; adapt progressively with treatment. - Limit to a maximum of 3–4 iterations; engrave for differentiation. - Thoroughly polish, especially the extension, to ensure smooth sliding and prevent mucosal irritation. - Do not alter prototype thickness to maintain stability. - Ensure no manufacturing defects (e.g., bubbles, microcracks, sharp edges); reprint if defects are present. - Prototype must fit securely on the model without rotation or wobbling.
	Endoscopic fit evaluation	- No feeding at least 2 h before evaluation. - Assessment to exclude other possible causes of UAO. - Airway must be visibly open with unrestricted epiglottic movements after TPP insertion. - The TPP extension must apply sufficient, evenly distributed pressure on the tongue and be correctly positioned sagittally. - The extension should reach the base of the tongue without being too long to restrict physiological movement. - The extension should be positioned centrally on the tongue to prevent asymmetrical bulging of the tongue (especially in asymmetrical patients). - Base plate must have a secure, rotation-free hold. - The cleft region must allow adequate space to support normal physiological movement. - Ensure correct endoscopic protocol: neutral head position, no TPP movement, clear view. - After prototype insertion, the tongue is positioned anteriorly and visible. - Physiological conditions of the pharyngeal mucosa. - Prototype thickness should be ≥3 mm, width ≥ half of original, excessive length can be trimmed bedside, and any sagittal adjustments or lengthening must be done in the dental laboratory.
**Final TPP**	General	- No bubbles, micro-cracks, or sharp edges; regrind/re-polymerize if needed.
	Safety Wire	- Must be centred, without bubbles and fully polymerized; starts at the middle of the plate and reaches almost the extension’s end.
	Base Plate	- Ensure base plate has a secure fit on model; no rotation or tilting movement. - Sufficient vestibular extension to counterbalance forces. - Ensure sufficient spacing to allow natural movement of labial and buccal frenula. - Posterior curve of the base plate and narrowed top of extension reduce appearance of OMLs in that region and aid feeding.
	Extension	- Ensure minimum 2.5–3 mm thickness for stability (0.5 mm less than the prototype is possible due to the safety wire). - The velopharyngeal extension provides enough space for physiological movements, e.g., swallowing.
	Extraoral Fixation Bows	- Wires centred, rounded ends, shaped to accommodate the lip. - Fit properly from canine to cheek with adequate spacing.
	Final fitting on patient	- Monitor infant closely (e.g., retching, major irritation or restlessness, work of breathing, etc.). - Safety wire must always be in place, except ≤5 min during endoscopy.
**Follow-up**	Evaluation	- Assess fit, feeding, and ventilation regularly. - Inspect OMLs or vestibular dents. - Address OMLs early; polish, grind, or remake plate if needed. - Vestibular/alveolar dents are acceptable if non-inflammatory. - Breathing sounds and evaluation of sleep studies.
	Caregiver Training	- Teach correct device use and encourage regular mucosal inspection.
	Regular Monitoring	- Outpatient visits to detect OMLs early and adapt the appliance as needed.

**Table 2:** Summary of other centres with similar approaches to the TPP.

Author	Country	Used Name	Article Type	Type of TPP
Ludwig et al. (2006) [46]	Germany	TPP	Case report	Dynamic extension with two rounded single rods for both prototype and final
Kochel et al. (2011) [47]	Germany	TPP	Review and methodology	Two options, dynamic and static extension. Dynamic by a so-called “omega” shaped twisting of a single rod orthodontic wire for dynamic changes. Alternatively, a static version uses a polymer extension but without safety wire.
Gerzanic et al. (2012) [48]	Austria	TPP	Case report	Dynamic extension with two rounded single rods for both prototype and final. Improperly designed bows—too short and poorly positioned—leave insufficient space hindering feeding.
Ho et al. (2019) [49]	China	PEBP	Technical note with case report	Dynamic polymer extension setup. Combination of two rounded single rods to angle the extension and an orthodontic expansion screw in the middle half of the extension to allow length adjustments
Tomic et al. (2020) [50]	Austria	MPP	Original article	Dynamic extension with two rounded single rods for both prototype and final. Substantially shorter length of extension compared to other approaches, possibly insufficient to relieve the UAO for most cases.
Schmidt et al. (2020) [51]	Germany	PEBP	Report	Dynamic extension with two rounded single rods for both prototype and final. Double wire for prototype and final. Extraoral retention bows extend from inside the plate and surround the vestibular rim, making bedside adjustment of the edge difficult or impossible (potential increasing risk of OMLs). Rods only in top portion of extension, do not run down the length of the extension.
Goryachkina et al. (2020) [52]	Russia	PEBP	Original article (*in Russian*)	Dynamic extension with two rounded single rods for both prototype and final
Thurzo et al. (2022) [26]	Slovakia	TPP	Original article	Proof-of-concept using CT for a completely 3D-printed prototype with wires.
Cho et al. (2022) [53]	USA	OAP	Case report	Rigid extension with no safety wire; only polymer transparent extension
Cho et al. (2022) [54]	USA/Korea	OAP	Brief report	Split plate with expansion screw in palatal plate + thermoplastic extension
Benitez et al. (2024) [55]	Switzerland	PEBP	Technical report	Dynamic polymer extension setup + double wire for prototype. Rigid final appliance (different safety wire)

**Table 3:** Resume on staged orthodontic management in patients with RS.

Dentition Stage	Age (Years)	Orthodontic Measures
Primary dentition	4–6	Early management of transverse maxillary deficiency and mandibular retrognathia using Class II functional appliances with expansion to guide mandibular growth. - Severe cases may require fixed palatal expanders - Mild cases: removable plates.
Early mixed dentition	8–9	- Reassessment of craniofacial growth and dentition - Continued use of functional appliances due to limited fixed anchorage.
Late mixed dentition	10–12	- Fixed Class II appliances address skeletal issues - Skeletal anchorage (e.g., Hyrax with mini-implants) enables transverse expansion - Common dentoalveolar issues include impacted/displaced teeth and tooth agenesis [63]; surgical exposure and individualized space management are often required.
Pubertal growth	♀: 10–14 ♂︎: 12–16	Functional appliances designed to utilize the pubertal growth spurt for guiding craniofacial growth in a physiological direction
After full permanent dentition eruption	Ca. 12	- Begin with multi-bracket appliances to align and level the teeth, coordinate the arches, correct malocclusion, and optimize occlusion and esthetics through controlled forces: - Expansion appliances and multibracket systems to achieve arch symmetry; extractions considered if needed.
Skeletal dysgnathia	End of skeletal growth	Severe skeletal dysgnathia may require corrective osteotomy in collaboration with maxillofacial surgery.

## Data Availability

Data are available from the corresponding author upon reasonable request. Information to patients is not available given the patient data protection law.

## Supplementary Materials

The following supporting information can be downloaded at: https://www.mdpi.com/article/10.3390/bioengineering12101063/s1, technical drawings and .stl files of the Tübingen standard velopharyngeal extensions; for both patients with and without cleft palate.

## Abbreviations

The following abbreviations are used in this manuscript:

RS	Robin Sequence
UAO	Upper Airway Obstruction
TPP	Tübingen Palatal Plate
PEBP	Pre-epiglottic Baton Plate
IOS	Intraoral Scanning
CAD/CAM	Computer-Aided Design and Computer-Aided Manufacturing
AM	Additive Manufacturing
OAI	Mixed-Obstructive Apnoea Index
STL	Standard Tessellation Language
PLY	Polygon File Format
DCM	Digital Imaging and Communications in Medicine
SLA	Stereolithography
IPA	Isopropyl Alcohol
DLP	Direct Light Processing
MDR	Medical Device Regulation
PMMA	Polymethyl methacrylate
MU	Mucosal Ulceration
OML	Oral Mucosal Lesion
MRI	Magnetic Resonance Imaging
CT	Computed Tomography
SM	Subtractive Manufacturing
PEEK	Polyetheretherketone
MPP	Modified palatal Plate
OAP	Orthodontic Airway Plate
MDO	Mandibular Distraction Osteogenesis
PAS	Posterior Airway Space
TMJ	Temporomandibular joint
CD	Craniofacial Disorder
ERN	European Reference Network
