# Supplementary material for: Complete Protocol and Guidelines for the Implementation and Manufacturing of the Tübingen Palatal Plate—An Interdisciplinary Technical Note on the Tübingen Approach for Infants with Robin Sequence
Source: Bioengineering (Basel). 2025 Sep 30;12(10):1063. doi: 10.3390/bioengineering12101063 (PMC12561442; doi:10.3390/bioengineering12101063)
Supplement: Supplementary file 1 [file bioengineering-12-01063-s001.zip › TPP_velopharyngeal extension designs_technical drawings.pdf]

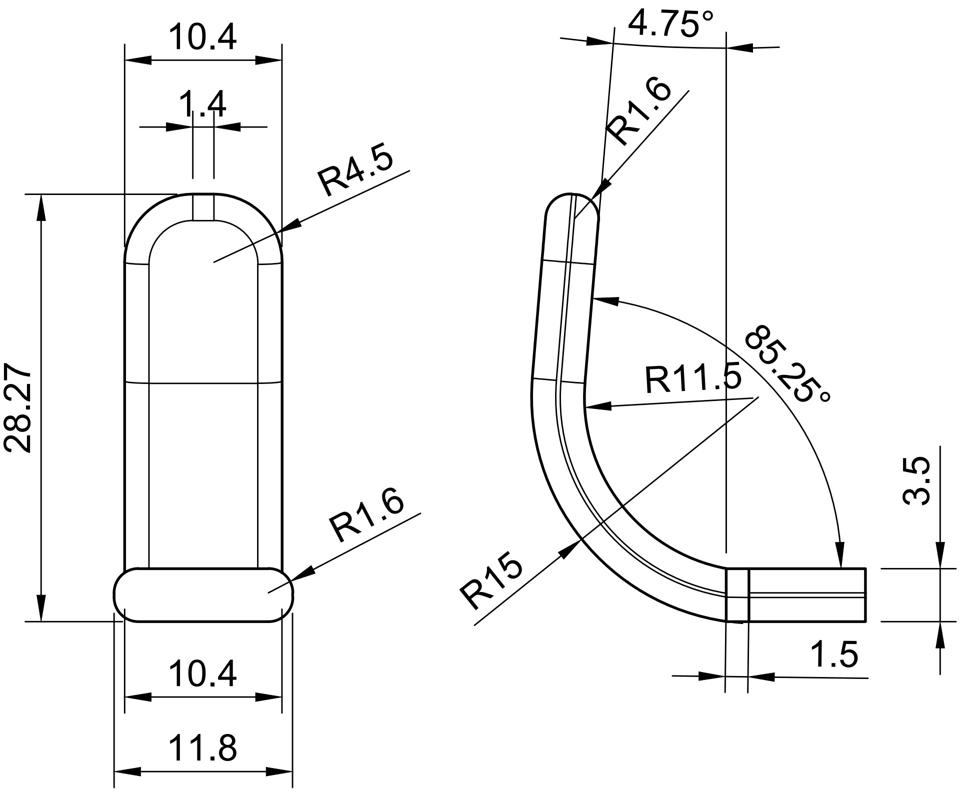

Dept.	Technical reference	Created by	Approved by
		Document type	Document status
		Title: Extension	DWG No.
			Rev. / Date of issue / Sheet 1/1

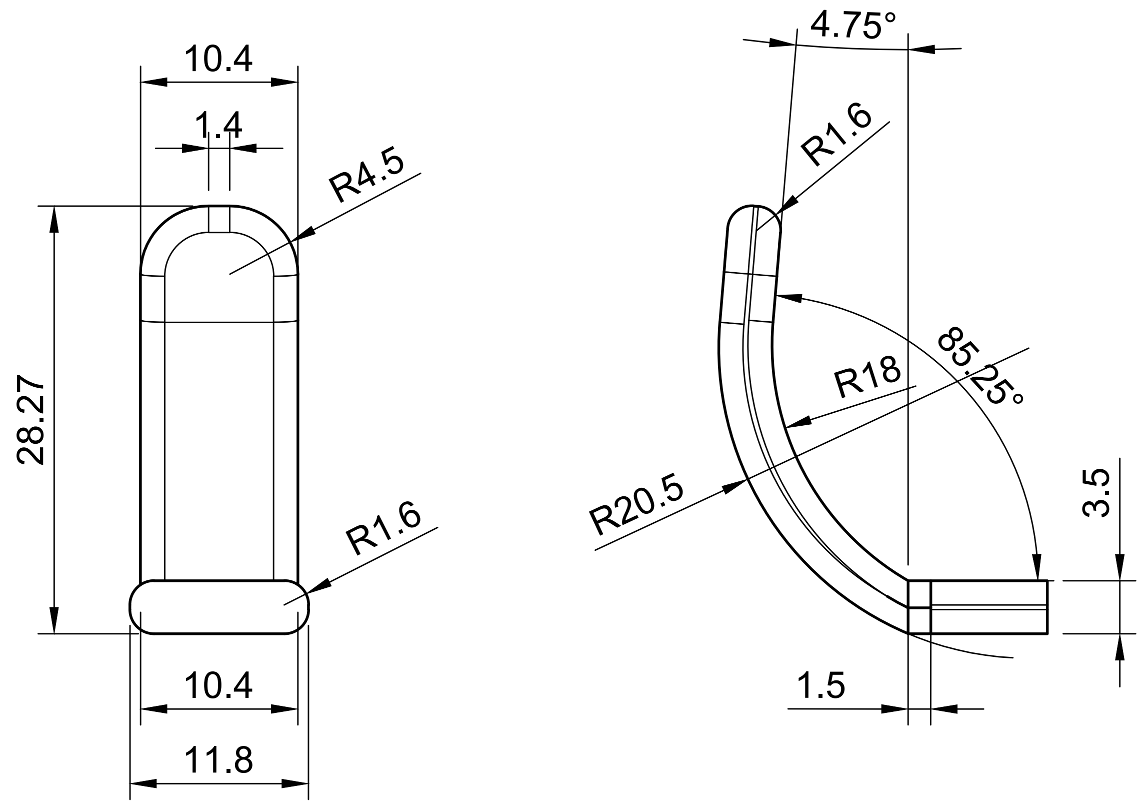

Dept.	Technical reference	Created by	Approved by
		Document type	Document status
		Title<br>Extension no cleft	DWG No.
			Rev. / Date of issue / Sheet 1/1
